# Indium-MOF as Multifunctional Promoter to Remove Ionic Conductivity and Electrochemical Stability Constraints on Fluoropolymer Electrolytes for All-Solid-State Lithium Metal Battery

**DOI:** 10.1007/s40820-025-01760-x

**Published:** 2025-05-07

**Authors:** Xiong Xiong Liu, Long Pan, Haotian Zhang, Cancan Liu, Mufan Cao, Min Gao, Yuan Zhang, Zeyuan Xu, Yaping Wang, ZhengMing Sun

**Affiliations:** School of Materials Science and Engineering, Southeast University, Nanjing, 211189 People’s Republic of China

**Keywords:** Fluoropolymer, Solid polymer electrolyte, Electrochemical stability, In-MOF, Solid electrolyte interphase, All-solid-state lithium metal battery

## Abstract

Fluoropolymers promise all-solid-state lithium metal batteries (ASLMBs) but suffer from two critical challenges. The first is the trade-off between ionic conductivity (*σ*) and lithium anode reactions, closely related to high-content residual solvents. The second, usually consciously overlooked, is the fluoropolymer’s inherent instability against alkaline lithium anodes. Here, we propose indium-based metal–organic frameworks (In-MOFs) as a multifunctional promoter to simultaneously address these two challenges, using poly(vinylidene fluoride–hexafluoropropylene) (PVH) as the typical fluoropolymer. In-MOF plays a trio: (1) adsorbing and converting free residual solvents into bonded states to prevent their side reactions with lithium anodes while retaining their advantages on Li^+^ transport; (2) forming inorganic-rich solid electrolyte interphase layers to prevent PVH from reacting with lithium anodes and promote uniform lithium deposition without dendrite growth; (3) reducing PVH crystallinity and promoting Li-salt dissociation. Therefore, the resulting PVH/In-MOF (PVH-IM) showcases excellent electrochemical stability against lithium anodes, delivering a 5550 h cycling at 0.2 mA cm^−2^ with a remarkable cumulative lithium deposition capacity of 1110 mAh cm^−2^. It also exhibits an ultrahigh *σ* of 1.23 × 10^−3^ S cm^−1^ at 25 °C. Moreover, all-solid-state LiFePO_4_|PVH-IM|Li full cells show outstanding rate capability and cyclability (80.0% capacity retention after 280 cycles at 0.5C), demonstrating high potential for practical ASLMBs.

**Supplementary Information:**

The online version contains supplementary material available at 10.1007/s40820-025-01760-x.

## Highlights

- Indium-based metal–organic framework (In-MOF) is proposed as a multifunctional promoter to create poly(vinylidene fluoride–hexafluoropropylene) (PVH)/In-MOF (PVH-IM) composite solid polymer electrolyte, synchronously achieving a high ionic conductivity of 1.23 × 10^−3^ S cm^−1^ and excellent electrochemical stability against Li anodes.
- In-MOF not only can adsorb and convert free residual solvents into bonded states to prevent their side reactions with Li anodes, but also induce inorganic-rich solid electrolyte interphase layers to prevent PVH from reacting with lithium anodes and promote uniform lithium deposition without dendrite growths.
- The Li|PVH-IM|Li symmetric cells maintain stable cycling for 5550 h at the current density of 0.2 mA cm^−2^. In addition, all-solid-state LFP|PVH-IM|Li full cells deliver a significant capacity retention of 80.0% at a rate of 0.5C after 280 cycles at 25 °C.

**Supplementary Information:**

The online version contains supplementary material available at 10.1007/s40820-025-01760-x.

## Graphical abstract

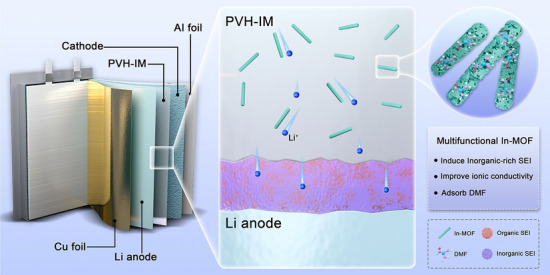

## Introduction

The energy density of traditional Li-ion batteries has approached their theoretical limits, and using liquid electrolytes has raised widespread safety concerns [1–4]. All-solid-state Li metal batteries (ASLMBs) are considered the "holy grail" of next-generation electrochemical energy-storage technologies due to their theoretically high energy density and intrinsic safety [5–7]. Solid-state electrolytes are one of the most fundamental components that determine the electrochemical performance of ASLMBs, requiring high ionic conductivity (*σ*), interfacial contact, electrochemical stability, high strength, *etc.* [8–10]. Among various candidates, solid-state polymer electrolytes (SPEs) stand out because they showcase excellent flexibility enabling decent interfacial compatibility with electrodes, good processability promising large-scale production, and tailored physical/chemical properties endowing multifunctionality [11, 12]. In particular, fluoropolymers provoke great attention because they have high dielectric constants (*ε*_*r*_ = 8–12) that significantly enhance the dissolution and dissociation of Li salts, resulting in higher *σ* compared to other SPEs [13–15]. In addition, fluoropolymers show excellent elasticity, mechanical strength (~ 50 MPa), and thermal stability (*T*_d_: 400 °C) [16, 17]. With these merits, fluoropolymers are promising for developing high-performance SPEs [18, 19]. However, fluoropolymers suffer from two critical problems, hindering their implementation in next-generation ASLMBs.

The first problem is the compromise between the *σ* and Li anode side reactions, which strongly correlate with residual solvents [20, 21]. Generally, solution casting is the most common approach for preparing fluoropolymer SPEs, which involves polar solvents such as *N*,*N*-dimethylformamide (DMF) and *N*-methylpyrrolidone (NMP) [22, 23]. These solvents have high polarity and boiling points, making them difficult to completely remove upon drying. As a result, fluoropolymer SPEs usually exhibit high residual solvent content of 10–17 wt% [24–26]. On the one hand, the residual solvents act as plasticizers and form complexes with Li^+^ (*e*.*g*., Li(DMF)_*x*_^+^), thereby accelerating Li^+^ migrations and enhancing *σ* to 10^−4^ S cm^−1^ [27–30]. On the other hand, the polar residual solvents thermodynamically and kinetically favor reacting with Li metal anodes [31, 32]. The side reactions continuously consume Li metal anodes and form thick solid electrolyte interphase (SEI) layers, significantly boosting interfacial impedances and speeding up battery failures. In other words, reducing residual solvent content diminishes the side reactions with Li metal anode but will inevitably decrease the *σ* to 10^−6^–10^−5^ S cm^−1^ [20]. Therefore, it is essential to minimize the side reactions of high-content residual solvents with Li metal anode, without compromising their beneficial effects on *σ*.

**Fig. 1:**
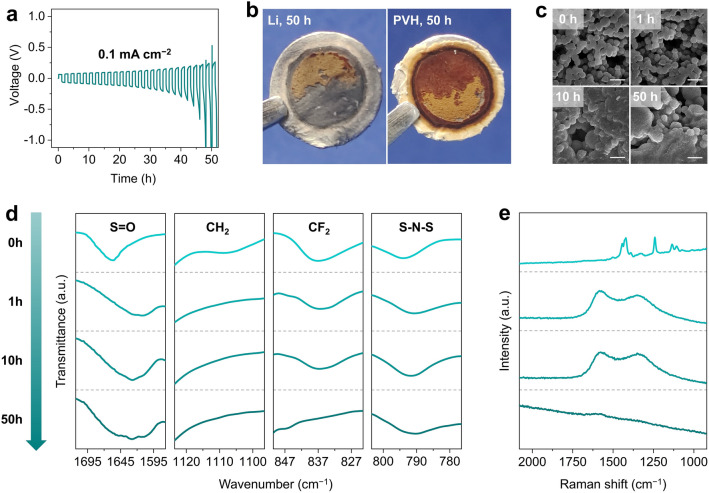
Electrochemical stability of PVH against Li metal anode. **a** Galvanostatic voltage profiles of Li|PVH|Li symmetric cells at 0.1 mA cm^−2^. **b** Photographs of PVH and Li metal after 50 h cycling at 0.1 mA cm^−2^. **c** Scanning electron microscopy **(**SEM) images, **d** Fourier transform infrared spectroscopy **(**FT-IR) profiles, and **e** Raman spectra of PVH after 0, 1, 10, and 50 h cycling at 0.1 mA cm^−2^. The scale bars in **c** are 5 μm. All tests are conducted at 25 °C unless otherwise stated

The second problem, which is much more critical but intentionally overlooked, is the unexplored stability of fluoropolymers against Li metal anodes. As is well known in organic chemistry, fluoropolymers usually showcase excellent chemical stability except for alkali chemicals [33, 34]. In this regard, they undergo dehydrofluorination under alkaline environments, leading to severe structure and performance degradation [35]. In this sense, Li, as a representative alkali metal, typically exhibits strong alkalinity, which will definitely influence the stability of fluoropolymers. Unfortunately, this problem has been neglected in ASLMBs to date. To reveal this phenomenon, we employ poly(vinylidene fluoride–hexafluoropropylene) (PVH), one of the most typical fluoropolymers, to assemble symmetric Li cells. They are only able to cycle for 50 h at a small current density of 0.1 mA cm^−2^ (Fig. 1a). After cycling, the Li foil loses metallic luster, and the PVH turns dark brown (Figs. 1b, S1). The PVH also sticks to Li foils, making them very difficult to separate. Moreover, the uniform PVH spheres gradually merge into large particles with inhomogeneous size distributions as cycling continues (Fig. 1c). The diffraction peaks of PVH also gradually disappear, indicating its structural degradation (Fig. S2a). Similar results are also observed in optical spectroscopy profiles, where the characteristic peaks of –CF_2_– and –CH_2_– vanish (Figs. 1d, S2b) with the emergence of distinct carbon bands (Fig. 1e). These changes imply the dehydrofluorination and the formation of amorphous carbon [36, 37]. Note that carbon is a good electron conductor, which causes continuous decomposition of PVH. Therefore, it is of great importance to optimize the chemical and electrochemical stability of PVH against Li metal anodes, with the combination of breaking the residual solvent-induced compromise between σ and Li anode side reactions.

Herein, we propose one-dimensional (1D) indium metal–organic frameworks (In-MOFs) as a multifunctional promoter to simultaneously address these challenges, resulting in a composite SPE involving PVH and In-MOF (labeled as PVH-IM). Note that the In-MOF is employed because of its highly porous structures, open metal sites, and 1D morphology [38, 39]. Specifically, the microporous and mesoporous structure of In-MOF is conducive to adsorbing and immobilizing the residual solvent (*i*.*e*., DMF in this case), thereby preventing side reactions between DMF and Li metal anodes while maintaining the capability to facilitate Li^+^ transport. In addition, compared to the other MOFs (*e*.*g*., Fe-MOF, Cr-MOF), the In-MOF exhibits the lowest adsorption energy (Fig. S3). Furthermore, the 1D morphology of In-MOF optimizes its interfacial contacts with the PVH matrix, allowing fast Li^+^ transport at the PVH/In-MOF interfaces [40]. More importantly, the In-MOF has a much smaller LUMO (*i*.*e*., lowest unoccupied molecular orbital) energy level compared to PVH, Li salt, and DMF. Accordingly, the surface In-MOFs faced to Li metal anodes are the first to react with Li metal and generate a thin uniform inorganic-rich SEI layer, which, in turn, prevents the side reactions between PVH and Li metal anodes.

As a result, the PVH-IM demonstrates significant cycling stability against Li metal anodes, delivering an ultralong cycle life of 5550 h at the current density of 0.2 mA cm^−2^, which overwhelms most previous fluoropolymer and other polymer-based SPEs. In addition, the PVH-IM exhibits a high critical current density (CCD) of 1.7 mA cm^−2^ and an excellent σ of 1.23 × 10^−3^ S cm^−1^ at 25 °C, outperforming most previous reports. Furthermore, the enhancement mechanism is comprehensively revealed using various experimental and theoretical techniques. Finally, when assembled with LiFePO_4_ (LFP) cathode and Li metal anode, the PVH-IM-based all-solid-state full cells demonstrate stable operation over 280 cycles at the current density of 0.5C under 25 °C, exhibiting an extraordinary capacity retention of 95.7%. This work sheds a bright future for accelerating the practical applications of high-performance fluoropolymer-based SPEs for next-generation ASLMBs.

## Results and Discussion

### Preparation and Characterization of PVH-IM

The synthesis of PVH-IM involves two steps (see details in the Experimental Section). Briefly, the first step contains the preparation of In-MOF using a previously reported method, which exhibits the 1D rod-like morphology and good crystallinity (Fig. S4) [38, 39, 41]. In the next step, PVH-IM was obtained by mixing PVH and In-MOF through a simple solution-casting method, as shown in Fig. S5. PVH was also prepared as contrast samples using the same procedure without adding In-MOF.

Figure S6 shows that the PVH displays interconnected microsphere morphology. After introducing In-MOF, the morphology of PVH remains unchanged, while the In-MOF nanorods are uniformly dispersed in the PVH matrix (Fig. S7). Additionally, the crystal structure of In-MOF does not alter in the PVH-IM, as XRD results show in Fig. S8, indicating that the preparation process is nondestructive. Moreover, incorporating In-MOF significantly decreases the crystallinity of PVH, indicating the increase in amorphous regions in the PVH matrix, which effectively facilitates Li^+^ transport (as discussed later) [19].

### Effect of In-MOF on Electrochemical Stability of PVH against Li Metal Anodes

**Fig. 2:**
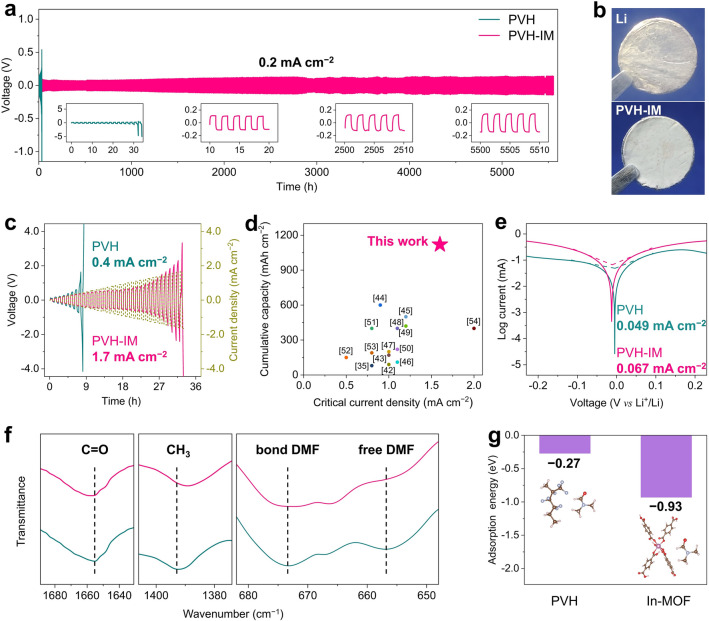
Electrochemical stability of PVH-IM against Li metal anode. **a** Galvanostatic voltage profiles of Li|PVH-IM|Li symmetric cells at 0.2 mA cm^−2^. **b** Photographs of PVH-IM and Li metal anode after 50 h cycles at 0.1 mA cm^−2^. **c** CCD curves of PVH and PVH-IM. **d** Comparison of the cumulative Li striping/plating capacity and CCD values of PVH-IM with other reported SPEs. **e** Tafel plots and corresponding exchange current densities of PVH and PVH-IM. **f** FT-IR spectra of PVH and PVH-IM. **g** Adsorption energies of PVH/DMF and In-MOF/DMF. To simplify the adsorption energy calculation, only one vinylidene fluoride unit and one hexafluoropropylene unit are used to represent the PVH chains. All tests are conducted at 25 °C unless otherwise stated

Li symmetric cells are assembled using Li foils and PVH-IM as electrodes and SPE, respectively, in order to verify the enhancement of In-MOF on PVH's electrochemical stability against Li metal anode. Figure 2a presents that the Li|PVH-IM|Li symmetric cells maintain stable cycling for 5550 h at the current density of 0.2 mA cm^−2^, delivering a remarkable cumulative Li stripping/plating capacity of 1110 mAh cm^−2^. In addition, the polarization voltage Li|PVH-IM|Li is small and keeps stable upon cycling. In contrast, the Li|PVH|Li cells fail quickly after only 34 h under the same current density (inset of Fig. 2a). Moreover, the Li|PVH-IM|Li cells exhibit stable operation for 500 h even at a high current density of 0.5 mA cm^−2^, while the Li|PVH|Li cells fail to work at all (Fig. S9). We subsequently observe the cycled PVH-IM and Li metal anodes by disassembling the Li|PVH-IM|Li cells. The metallic luster of Li metal anodes is well retained, and the white color of PVH-IM remains unchanged after 50 h cycling (Figs. 2b, S10). On the contrary, the PVH and Li metal anodes suffer significant degradation with noticeable color change in the case of cycled Li|PVH|Li cells (Fig. 1b). These results firmly demonstrate that In-MOF remarkably improves the electrochemical stability of PVH against Li metal anodes.

CCD is recorded to evaluate the maximum available current density of a SPE, as displayed in Fig. 2c. The PVH-IM exhibits a high CCD of 1.7 mA cm^−2^, which is 4.25 times that of PVH (0.4 mA cm^−2^). Note that the CCD and total Li stripping/plating capacity of our PVH-IM surpass those reported in previous studies (Fig. 2d) [35, 42–54]. Furthermore, Tafel tests are conducted to calculate the exchange current density. In general, a higher exchange current density indicates faster ion transport kinetics at the SPE/Li metal interface. Figure 2e shows that the exchange current density of PVH-IM is 0.067 mA cm^−2^, which is 1.37 times that of PVH (0.049 mA cm^−2^), demonstrating that In-MOF significantly promotes Li^+^ transport [25, 32].

The influence of In-MOF on the states of residual solvent DMF is firstly studied to reveal the reason for the excellent electrochemical stability of PVH-IM. Thermogravimetric (TG) analysis shows that the residual DMF content in PVH and PVH-IM is 14.9% and 14.3%, respectively, with a negligible difference (Fig. S11). As mentioned above, the residual DMF improves σ but reacts seriously with Li metal anodes. FT-IR is used to characterize the form of residual DMF (Fig. 2f). In the case of PVH, the two characteristic peaks at 1655.1 and 1392.4 cm^−1^ correspond to the C=O and CH_3_ groups of DMF. These two peaks shift to 1657.5 and 1389.0 cm^−1^ after introducing In-MOF, indicating the strong interactions between In-MOF and DMF. In addition, the PVH shows a characteristic peak of free DMF molecules at 656.6 cm^−1^, which disappears in the case of PVH-IM [31, 55, 56]. These findings imply that the overwhelming majority of free DMF molecules are bonded by interacting with In-MOF.

Subsequently, we conduct density functional theory (DFT) calculations to confirm the strong interaction between In-MOF and residual DMF molecules by calculating the adsorption energy of DMF with PVH and In-MOF. As shown in Fig. 2g, the adsorption energy of DMF with PVH chain is −0.27 eV. In contrast, the adsorption energy of DMF with In-MOF is as low as −0.93 eV, indicating the strong adsorption DMF capability of In-MOF. In addition, the distance between two In-ion chains of In-MOF is 10.89 Å, which is significantly larger than the molecular diameter of DMF (1.74 Å), making it structurally possible to absorb DMF molecules [31, 38, 39]. As a result, the strong adsorption of In-MOF to DMF minimizes the side reactions between residual DMF and Li metal anodes, contributing partially to the excellent enhancements in the electrochemical stability of PVH-IM.

### Mechanism of In-MOF to Protect PVH from Reacting with Li Metal Anodes

**Fig. 3:**
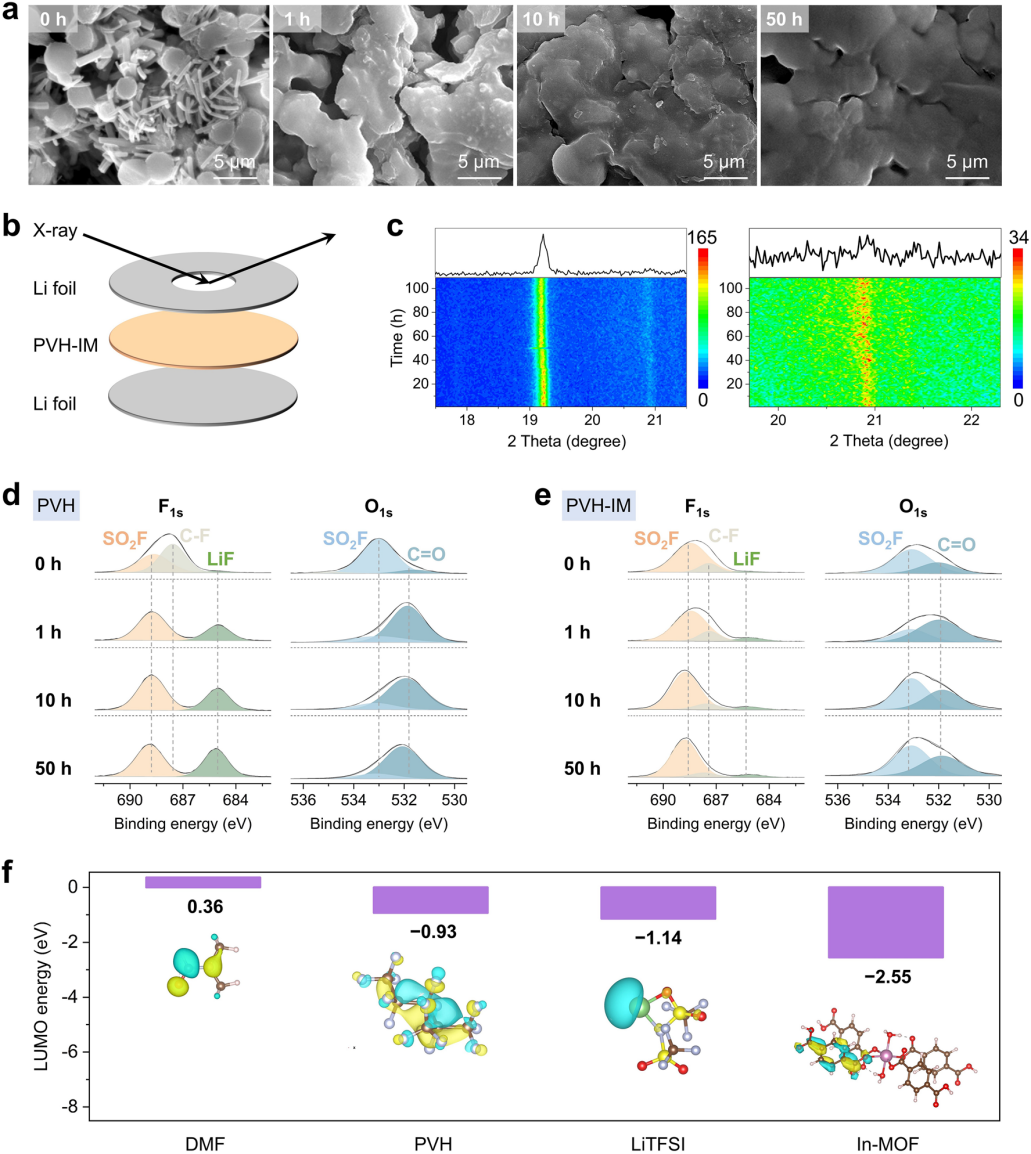
Characterizations of cycled PVH-IM. a SEM images of PVH-IM after 0, 1, 10, and 50 h cycling at 0.1 mA cm^−2^. **b** Schematic and **c** corresponding patterns of the in situ XRD measurements of PVH-IM. XPS spectra of **d** PVH and **e** PVH-IM after 0, 1, 10, and 50 h cycling at 0.1 mA cm^−2^. **f** LUMO energy diagrams of DMF, PVH, LiTFSI, and In-MOF. To simplify the LUMO energy calculation, the numbers of vinylidene fluoride units and hexafluoropropylene units in the PVH molecular chains are fixed as one

In addition to suppressing side reactions between residual DMF and Li metal anodes, the In-MOF also partially sacrifices to form a thin yet robust SEI layer, thereby isolating PVH and Li metal anode to prevent their inherent reactions. To demonstrate this hypothesis, we first perform various experimental characterizations and theoretical simulations on the PVH-IM side after cycling, as illustrated in Fig. S12. SEM is used to observe the morphological changes of PVH-IM upon cycling (Fig. 3a). After 1 h cycling, part of the In-MOF nanorods on the surface disappear. In addition, the PVH microspheres tend to merge, resulting in increased PVH diameter and smaller pores. As the cycling continues, the In-MOF nanorods on the surface disappear entirely, while the microsphere morphology of PVH is retained. The disappearance of In-MOF on the surface is attributed to its reactions with Li metal anodes, generating a thin, uniform, and robust inorganic-rich SEI layer (as proved later). This inorganic-rich SEI layer acts as an ion-conducting binder to connect PVH microspheres and form a smooth and dense surface, resulting in tight PVH-IM/Li contact and homogenous Li deposition. This inorganic-rich SEI layer also serves as an electron isolator to efficiently prevent PVH from reacting with Li metal anodes. In contrast, the PVH undergoes severe reactions against Li metal anodes without In-MOF, resulting in uneven and rough surfaces after cycling (Fig. 1c), which is unfavorable for its contact with Li metal anode and uniform Li deposition.

The disappearance of In-MOF on the surface and the preservation of PVH crystal structure in the case of PVH-IM are further confirmed by in situ X-ray diffraction (XRD). To enable X-ray penetration through the lithium metal symmetric cell, a small hole is drilled in the center of the Li foil, as schematically illustrated in Fig. 3b. The corresponding in situ XRD patterns are plotted in Fig. 3c. The pronounced peak at 19.2° corresponds to the In-MOF, which shows decreased intensity upon cycling [38, 39]. This result indicates that only part of In-MOF nanorods are reacted and converted, leading to decreased peak intensity. At the same time, the weak peak around 20.9°, which is attributed to PVH, remains almost unchanged in intensity [27]. This result shows that the structure and crystallinity of PVH are not destroyed upon cycling. The in situ XRD findings are also verified by ex situ FT-IR and Raman analyses, where the characteristic peaks of In-MOF gradually disappear, and the characteristic peaks of PVH remain unaltered, as shown in Figs. S13 and S14. In contrast, in the case of PVH without In-MOF, the PVH’s characteristic peaks vanish, and new phases (such as carbon) emerge as cycling goes on (Fig. 1d, e). In addition, the cross-sectional SEM images of PVH-IM after 0, 1, 10, and 50 h cycling at 0.1 mA cm^−2^ are shown in Fig. S15. The 1D rod-like In-MOFs are still visible in the PVH-IM. These results demonstrate that the In-MOF on the PVH-IM surface preferentially reacts with Li metal anodes during cycling, thus protecting the PVH matrix from degradation.

Postmortem XPS examinations are subsequently employed to reveal the reaction products between SPEs and Li metal anodes. In the case of PVH (Fig. 3d), the F 1*s* spectrum shows that the peak of C-F bonds gradually disappears, suggesting intensive side reactions of PVH and LiTFSI with Li metal anodes as the cycling goes on. The side reactions lead to the gradual formation and increased contents of LiF, SO_2_F, and C=O on the surface of PVH [25, 27]. In contrast, the peak intensity of C-F bonds remains slightly changed, with a small broad LiF peak appearing. In addition, the contents of LiF, SO_2_F, and C=O are stable upon cycling (Fig. 3e). These findings imply that the In-MOF on the surface acts as a sacrificial agent to react with Li metal anodes, thereby protecting the PVH matrix from the attack of alkaline Li.

We also perform molecular orbital calculations to theoretically unravel the favorable reaction thermodynamics between In-MOF and Li metal anodes. Generally, the lowest unoccupied molecular orbital (LUMO) level represents electron-accepting properties, associating the resistance to reduction [57]. Figure 3f shows that In-MOF exhibits a LUMO energy of −2.55 eV, which is significantly smaller than those of DMF (0.36 eV), PVH (−0.93 eV), and LiTFSI (−1.14 eV). This result indicates that In-MOF has a strong tendency to accept electrons and react with Li metal anode at low potentials, thus forming a stable inorganic-rich SEI layer to prevent the reaction between PVH matrix and Li metal anodes.

**Fig. 4:**
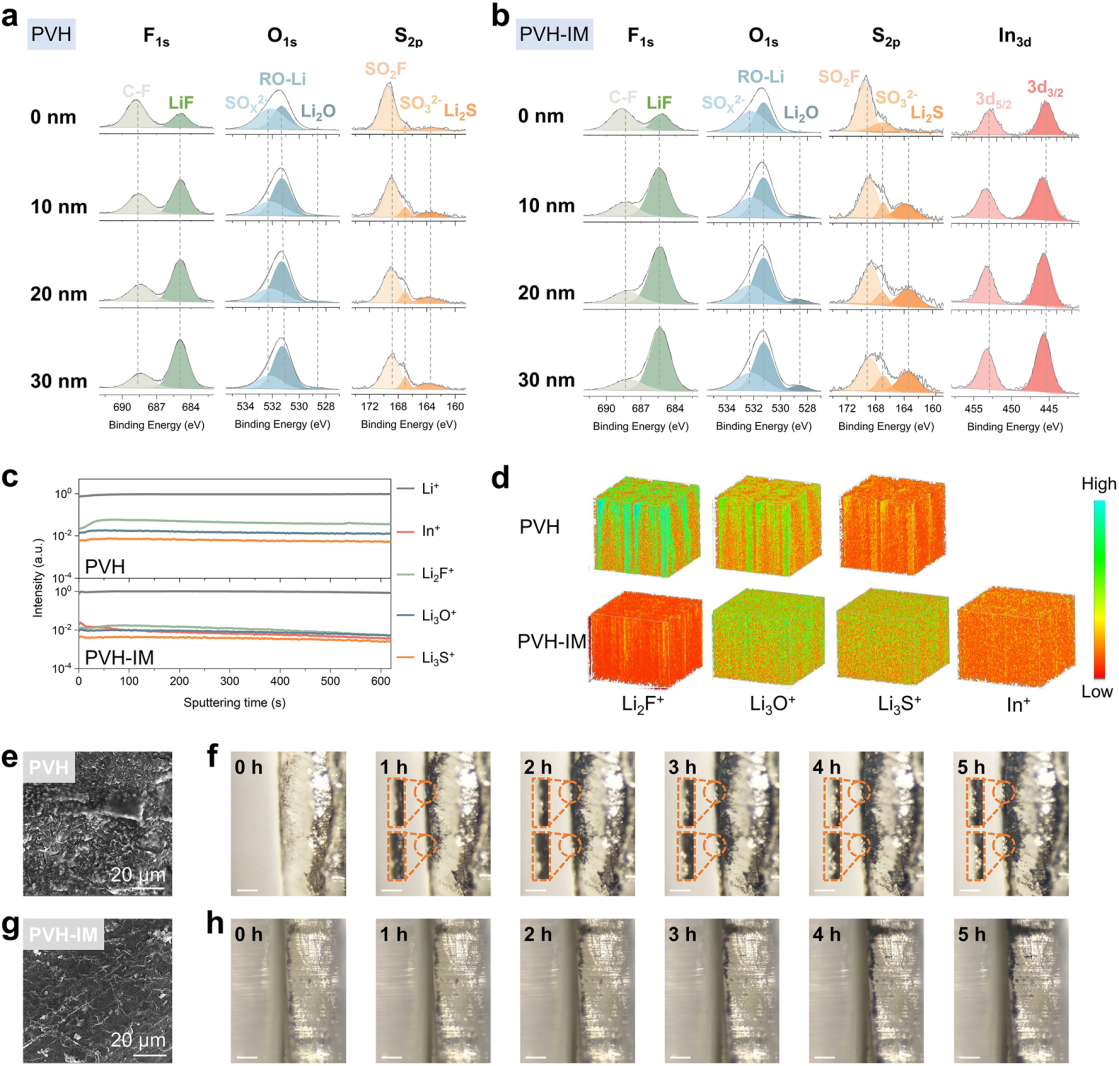
Characterizations of cycled Li metal anodes. Ar^+^-sputtering XPS profiles of SEI layers on Li metal surfaces from **a** Li|PVH|Li and **b** Li|PVH-IM|Li cells after cycling for 50 h at 0.1 mA cm^−2^. ToF–SIMS results of SEI layers on Li metal surfaces from Li|PVH|Li and Li|PVH-IM|Li cells after cycling for 50 h at 0.1 mA cm^−2^: **c** depth profiles and **d** 3D reconstruction images. SEM images of Li metal anode surfaces from **e** Li|PVH|Li and **g** Li|PVH-IM|Li cells after cycling for 50 h at 0.1 mA cm^−2^. In situ optical microscope images of **f** Li|PVH|Li and **h** Li|PVH-IM|Li cells during Li plating. The scale bars in **f** and **h** are 1 mm

In addition to the characterizations on the PVH-IM side after cycling, we also conduct various investigations on the cycled Li metal anode side (as illustrated in Fig. S12), in order to disclose the inorganic-rich SEI layer induced by In-MOF. The in-depth XPS test was first used to characterize the chemical composition of the SEI layers, and the corresponding spectra are depicted in Fig. 4a, b. In the F 1*s* spectra, the peak at 688.4 eV corresponds to the organic C-F bonds, and the peak at 685.4 eV is attributed to the inorganic LiF [31, 32]. In both cases of PVH and PVH-IM (Fig. S16a), as the etching lasts (*viz.*, etching depth rises), the content of organic C-F bonds decreases while the content of inorganic LiF increases. This result implies that the inorganic LiF mainly distributes in the deep SEI layer. In addition, the SEI layer of PVH-IM has higher inorganic LiF ratio than that of PVH-IM at all etching depths. For instance, at an etching depth of 10 nm, the LiF content in PVH-IM is 73.3%, whereas in PVH, it is only 57.2% (Fig. S15a). Similar findings are also observed for the O 1*s* and S 2*p* spectra, where the SEI layer of PVH-IM shows a higher content of Li_2_O and Li_2_S when compared to PVH (Fig. S16b, c) [19, 47]. Moreover, intensive peaks attributed to the In-containing inorganic species are observed in PVH-IM (Fig. 4b), which does not appear in PVH. The In 3*d* peaks on the SEI surface at 452.9 and 445.5 eV are attributed to In 3*d*_5/2_ and In 3*d*_3/2_, respectively, and their positions align with those in In-MOF (Fig. S17). As the etching depth increases, these peaks shift toward higher binding energies. This phenomenon indicated a decrease in In electron density in the deep SEI layer, which is likely due to the transformation of In from its original coordination environment into fluorides, oxides, sulfides, etc., resulting in electron loss.

It is worth noting that the inorganic components are ion conducting but electron insulating. Besides, they have higher mechanical strength and modulus than their organic counterparts [48]. Therefore, in the cases of PVH-IM, the inorganic-rich SEI layers play three critical roles: (1) inhibiting side reactions between PVH and Li metal anodes by preventing electron conduction, (2) inducing uniform Li deposition by allowing fast Li^+^ transport across the electrolyte/Li interfaces, and (3) suppressing Li dendrite growth. In contrast, in the case of PVH, the organic-rich SEI layer fails to prevent the side reactions between PVH and Li metal anode, resulting in uneven Li deposition and Li dendrite growth.

Time of flight secondary ion mass spectrometry (ToF–SIMS) with Cs^+^ ion beam is also employed to further characterize the components and distribution of SEI layers. The depth profile in Fig. 4c shows that all signals of PVH remain stable even after 600 s of sputtering, while the signals of PVH-IM remain stable in the first 100 s and then gradually decrease over the sputtering time. These results suggest that the SEI layer of PVH is much thicker than that of PVH-IM, leading to a fast increase in interfacial resistance and cell failure of the PVH during cycling [20]. Figure 4d presents 3D reconstruction images. In the case of PVH, the LiF, Li_2_O, and Li_2_S-based species are unevenly distributed along the thickness direction of the SEI layer, forming band-like patterns. This result suggests that the SEI layer of PVH is thick and inhomogeneous. On the contrary, in the case of PVH-IM, these signals are uniformly distributed in the near-surface areas along the thickness direction of the SEI layer and then gradually weaken, demonstrating that a thin but uniform SEI layer is induced by In-MOF.

Based on the above investigations on the composition and distribution of SEI layers, their impacts on Li stripping/plating behavior are disclosed. Figure 4e observes massive Li dendrites with moss-like morphologies on the Li foils that are cycled with PVH, which is likely due to the uneven Li deposition caused by the inhomogeneous organic-rich SEI layers. To further confirm this phenomenon, we use optical microscopes to in situ observe the Li deposition in Li|PVH|Li symmetric cells and the results are displayed in Fig. 4f. As the Li plating goes on, distinct dendrites form and grow into large particles with sub-millimeter size. In addition, the gap between PVH and Li metal anode becomes more and more noticeable, indicating uneven Li deposition deteriorated interfacial contact. In contrast, no obvious Li dendrites are witnessed on the Li metal anode surface after cycling in the case of Li|PVH-IM|Li (Fig. 4g), which shows an ordered Li deposition pattern similar to fresh Li foils (Fig. S18). Also, no Li dendrites and interfacial gap are found during the in situ optical observation (Fig. 4h). These results confirm the uniform Li stripping/plating in the Li|PVH-IM|Li cells, resulting from the thin homogeneous inorganic-rich SEI layer induced by In-MOF.

**Fig. 5:**
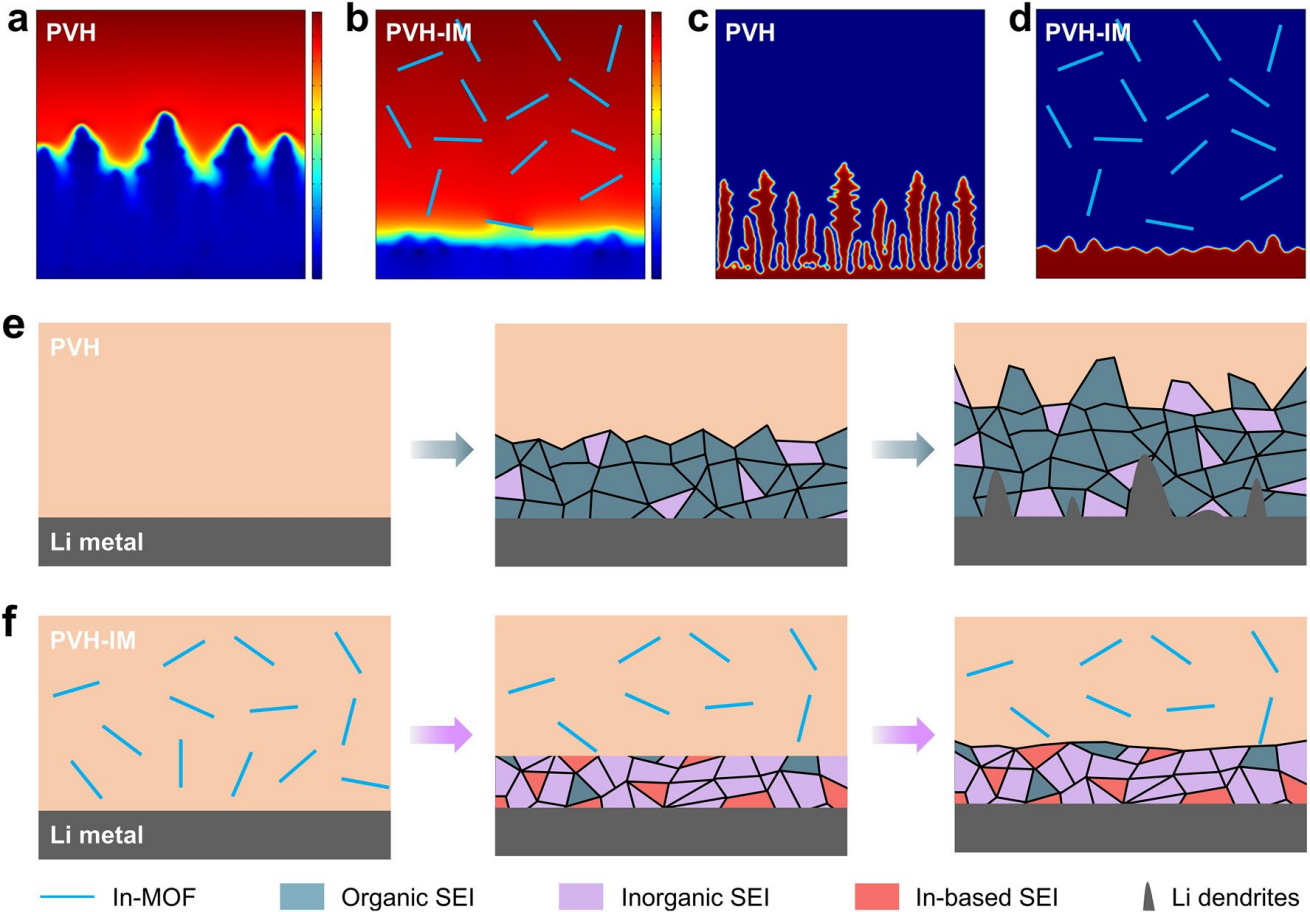
COMSOL Multiphysics simulations and SEI formation schematics. Simulated Li^+^ concentration distribution in **a** PVH and **b** PVH-IM. Simulated Li dendrite growth in **c** PVH and **d** PVH-IM. Schematics of SEI formation in **e** PVH and **f** PVH-IM. The simulation time of COMSOL Multiphysics is 150 s

To further unveil the Li deposition behaviors, we use COMSOL Multiphysics to simulate the Li dendrite growth on the Li metal anode surfaces with PVH and PVH-IM for a duration of 150 s. Figure 5a shows the Li^+^ concentration distribution of PVH, in which pronounced Li^+^ concentration gradients are observed at the tips. In this circumstance, Li^+^ tends to deposit at the tips, causing intensive Li dendrite propagation (Fig. 5c) [57]. After introducing In-MOF, the Li^+^ concentration is uniformly distributed, significantly reducing the tip concentration gradient and suppressing the tip effect (Fig. 5b) [57]. As a result, only a few small Li dendrites are formed in the case of PVH-IM (Fig. 5d). The COMSOL results again validate that introducing In-MOF nanorods promotes a smooth and uniform Li deposition on the Li metal surface and effectively avoids Li dendrite growth.

Based on the above-mentioned experimental and simulation findings, we summarize the effect of In-MOF on the PVH's electrochemical stability against Li metal anodes as follows (Fig. 5e, f). First, the In-MOF nanorods adsorb the residual DMF molecules, making them from free states to bonded states and suppressing their side reactions with Li metal anodes. Second, the In-MOF nanorods on the electrolyte surface serve as a sacrificial agent to preferentially react with Li metal anodes, forming a thin, uniform, and inorganic-rich SEI layer. This SEI layer not only protects the PVH matrix from reacting with Li metal anodes but also inhibits Li dendrite growth by inducing fast and uniform Li deposition. In contrast, in the case of PVH, the free residual DMF molecules and PVH matrix undergo intense side reactions with Li metal anodes, resulting in a thick, inhomogeneous, and organic-rich SEI layer that induces uneven Li deposition and Li dendrite formation.

### Ion-Conducting Properties of PVH-IM

**Fig. 6:**
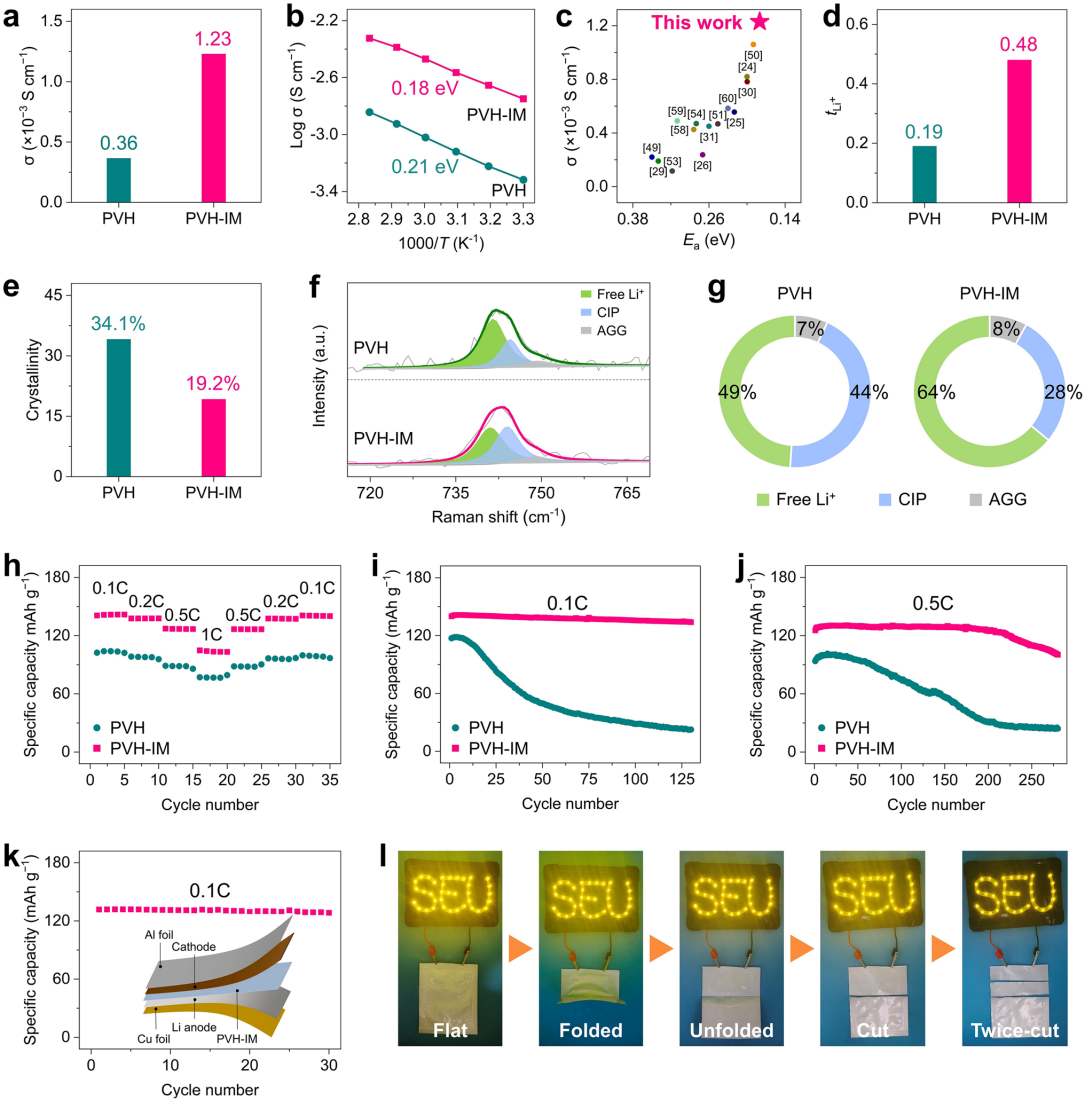
Ion-conducting behavior and all-solid-state full cell performance. **a** Ionic conductivities and **b** Arrhenius plots and corresponding activation energies of PVH and PVH-IM. **c** Comparison of *σ* and *E*_a_ of PVH-IM with previously reported SPEs [24–26, 29–31, 49–51, 53, 54, 58–60]. **d** $\it {\text{t}}_{{\text{Li}}^{+}}$, **e** crystallinities, **f** Raman spectra, and **g** contents of free TFSI.^−^, CIP, and AGG of PVH and PVH-IM. **h** Rate capability at different current densities and cycling stability at the current density of **i** 0.1C and **j** 0.5C of all-solid-state LFP|PVH|Li and LFP|PVH-IM|Li full cells. **k** Cycling performance of all-solid-state LFP|PVH-IM|Li pouch cells at the current density of 0.1C. **l** Photographs showing LED bulbs powered by all-solid-state LFP|PVH-IM|Li pouch cells under different testing states: flat, folded, unfolded, and cut. All tests are conducted at 25 °C unless otherwise stated. The crystallinities in **c** are calculated from the corresponding DSC curves in Fig. S22

In addition to its high electrochemical stability against Li metal anodes, PVH-IM also exhibits outstanding ion-conducting properties. Figure 6a presents that PVH exhibits an *σ* of 0.36 × 10^−3^ S cm^−1^ at 25 °C, which is significantly increased to 1.23 × 10^−3^ S cm^−1^ after introducing In-MOF. At the same time, the activation energy (*E*_a_) decreases from 0.21 eV for PVH to 0.18 eV for PVH-IM (Figs. 6b, S19). These values surpass most previously reported SPEs, as compared in Fig. 6c [24–26, 29–31, 49–51, 53, 54, 58–60]. The DC polarization was employed to measure the electronic conductivity (Fig. S20). The PVH-IM displays an electronic conductivity of 1.80 × 10^−9^ S cm^−1^, which is lower than that of PVH (9.13 × 10^−7^ S cm^−1^). Therefore, the improvement of ionic conductivity is not attributed to the electronic conductivity. In addition, Figs. 6d and S21 demonstrate that the PVH-IM exhibits a high Li^+^ transference number ($\it {\text{t}}_{{\text{Li}}^{+}}$) of 0.49, which is much larger than that of PVH (0.19). These results indicate that the presence of In-MOF effectively accelerates Li^+^ transport with a low energy barrier [61, 62].

To disclose the reasons for ion transport enhancement by In-MOF, we employ DSC and Raman to investigate the crystallinity change of the PVH matrix and the dissociation degree of LiTFSI, respectively. As Figs. 6e and S22 present, PVH showcases a high crystallinity of 34.1%, which dramatically declines to 19.2% in the case of PVH-IM. The reduced crystallinity suggests that introducing In-MOF brings about more amorphous regions in the PVH matrix, which is conducive to fast Li^+^ transport [63, 64]. In addition, our previous work demonstrated that Li^+^ is thermodynamically and kinetically favorable to migrate to and transport at the polymer/filler interfaces, suggesting that the fast-ion-conducting behaviors of PVH-IM also come from the accelerated Li^+^ conduction at the PVH/In-MOF interfaces [65].

Figure 6f, g displays the Raman spectra and their quantified analyses on LiTFSI dissociation. In general, there are three TFSI^−^ forms, including free TFSI^−^, contact ion pairs (CIP), and aggregated ion pairs (AGG), corresponding to the Raman bands at 740.9, 744.2, and 748.7 cm^−1^, respectively [63, 66]. Among them, the free TFSI^−^ represents the LiTFSI dissociation degree, which undergoes an enormous increase from 49% to 64% after introducing In-MOF nanorods, thereby showing a considerable contribution to improving σ. Furthermore, Lewis acid–base interactions between In-MOF and the Li salt immobilize the TFSI^−^ anions, resulting in improved $\it {\text{t}}_{{\text{Li}}^{+}}$ [48]. As a result, TFSI^−^ anions will not accumulate on the one side of electrolytes and deplete on the other side, thereby immensely reducing the space charge layer effects from and inhibiting the growth of Li dendrites [67, 68].

### All-Solid-State Full Cell Performance of LFP/PVH-IM/Li

Linear sweep voltammetry (LSV) is employed to investigate the oxidative stability of PVH-IM. Figure S23 shows that the current rises at approximately 4.10 V and then reaches a small but distinct oxidation peak at *ca*. 4.30 V in the case of PVH, suggesting its poor oxidation resistance at high voltages. Previous works also observe this peak but with a much smaller current intensity [63]. In contrast, the PVH-IM remains stable up to 4.90 V with almost zero current, indicating its excellent anti-oxidation capability at high voltages, which is important for practical applications for next-generation ASLMBs [69–71].

We then assemble all-solid-state full cells using LiFePO_4_ (LFP) as the cathodes, PVH and PVH-IM as solid-state electrolytes, and Li foil as the anodes. No liquid electrolytes or ionic liquids are added to the full cells. All cells are tested at 25 °C. Figure 6h presents the rate performance of all-solid-state full cells at different current densities. LFP|PVH-IM|Li delivers high reversible specific capacities of 141.7, 137.8, 126.9, and 103.5 mAh g^−1^ at the current density of 0.1C, 0.2C, 0.5C, and 1C, respectively. Once the current density returns to 0.5C, 0.2C, and 0.1C, the specific capacity immediately recovers to its initial values. On the contrary, LFP|PVH|Li exhibits much lower specific capacities at each current density than LFP|PVH-IM|Li. For instance, LFP|PVH|Li only delivers small specific capacities of 103.4 and 76.7 g^−1^ at the current density of 0.1C and 1C, respectively. In addition, LFP|PVH-IM|Li shows higher Coulombic efficiency than LFP|PVH|Li, confirming its excellent capacity reversibility (Fig. S24) [72, 73]. Moreover, the galvanostatic charging/discharging (GCD) profiles imply that LFP|PVH-IM|Li has lower polarization voltages than LFP|PVH|Li at all current densities (Figs. S25, S26), once again demonstrating the excellent ion conducting and electrochemical stability of PVH-IM.

Figure 6i presents the cycling performance of LFP|PVH-IM|Li and LFP|PVH|Li at the current density of 0.1C. After 130 cycles, LFP|PVH-IM|Li maintains a high specific capacity of 133.9 mAh g^−1^, corresponding to an excellent capacity retention of 95.7%. In contrast, the specific capacity of LFP|PVH|Li quickly decreases after the 7th cycle and finally reaches only 22.6 mAh g^−1^ at the 100th cycle. When the current density rises to 0.5C, the specific capacity of LFP|PVH-IM|Li shows almost no decline after 280 cycles with a high capacity retention of 80.0% (Fig. 6j). However, the specific capacity of LFP|PVH|Li gradually drops to 25.8% of its initial value. In addition, the LFP|PVH-IM|Li full cells exhibit more stable Coulombic efficiency than that of LFP|PVH-IM|Li at the current densities of 0.1C and 0.5C, demonstrating their excellent capacity reversibility (Fig. S27). These results demonstrate the outstanding cyclability of LFP|PVH-IM|Li.

We further assemble all-solid-state LFP|PVH-IM|Li pouch cells to verify the potential of PVH-IM for practical applications. The pouch cells demonstrate a high initial specific capacity of 131.8 mAh g^−1^ at the current density of 0.1C and undergo stable cycling for 30 cycles with a high capacity retention of 94.7% (Figs. 6k, S28). The pouch cells also successfully light up 36 light-emitting diodes (LEDs) (Fig. 6l). The LEDs remain powered when the pouch cells are folded and unfolded back and forth, suggesting their good flexibility. Even cut into several small pieces, the pouch cell continues to function properly without fuming or burning, demonstrating their high safety under extreme conditions.

To investigate the compatibility of PVH-IM with high-voltage cathode materials, the LiNi_0.6_Co_0.2_Mn_0.2_O_2_ (labeled as NCM622) was employed as a typical nickel-rich oxide cathode. The rate and cycling performance of NCM622|PVH-IM|Li full cells are shown in Fig. S29a, b. The all-solid-state NCM622|PVH-IM|Li full cells show excellent rate performance with high reversible specific capacities of 142.0, 132.1, 115.0, and 100.9 mAh g^−1^ at 0.1C, 0.2C, 0.5C, and 1C, respectively. In addition, the NCM622|PVH-IM|Li full cells exhibit a high initial specific capacity of 140.0 mAh g^−1^, which still maintains 138.5 mAh g^−1^ after 50 cycles at the current density of 0.1C under 25 °C (Fig. S29c, d). These results suggest that the PVH-IM holds excellent potential for various cathodes toward all-solid-state Li metal batteries.

## Conclusions

By proposing In-MOF as a multifunctional promoter, we have successfully overcome the poor electrochemical stability against Li metal anodes and the trade-off between residual solvent and σ for PVH. The intrinsic porous In-MOF enables efficient adsorption of residual DMF solvent, making them from free states into bonded states through interaction, thereby suppressing their side reactions with Li metal anodes. The In-MOF on the electrolyte surface also serves as a sacrificial agent to preferentially react with Li metal anodes, owing to its ultralow LUMO energy level. As a result, on the PVH-IM side, the reaction products act as a binder to bond PVH microspheres together, making the PVH-IM surface smoother and denser. The improved surface facilitates excellent interfacial contact between PVH-IM and Li metal anodes, promoting uniform Li deposition. On the Li metal anode side, In-MOF induces the formation of a thin, uniform, inorganic-rich SEI layer, which not only isolates the PVH matrix from Li metal anodes to avoid their reactions but also promotes uniform Li deposition to hinder Li dendrite growth. Moreover, In-MOF significantly reduces the crystallinity of the PVH matrix and promote LiTFSI dissociation. Consequently, the PVH-IM-based symmetric Li cells work properly for 5500 h at 0.2 mA cm^−2^, delivering a remarkable cumulative Li stripping/plating capacity of 1110 mAh cm^−2^. In addition, the PVH also achieves an excellent CCD of 1.7 mA cm^−2^, *σ* of 1.23 × 10^−3^ S cm^−1^, and *E*_a_ of 0.18 eV at 25 °C. Owing to these merits, the all-solid-state LFP|PVH-IM|Li full cells demonstrate outstanding rate capability (103.5 mAh g^−1^ at 1C) and cycle performance (95.7% and 80.0% capacity retention after 130 and 280 cycles at 0.1C and 0.5C, respectively) at 25 °C. Furthermore, the all-solid-state LFP|PVH-IM|Li pouch cells operate stably at different bending states and remain safe under extreme conditions. This work provides a facile and effective strategy to develop advanced fluoropolymer-based SPEs with both high σ and excellent stability against Li metal anodes, paving the way for the practical application of ASLMBs.

## Supplementary Information

Below is the link to the electronic supplementary material.

Supplementary file1 (DOCX 10999 KB)
