# Supplementary material for: Indium-MOF as Multifunctional Promoter to Remove Ionic Conductivity and Electrochemical Stability Constraints on Fluoropolymer Electrolytes for All-Solid-State Lithium Metal Battery
Source: Nanomicro Lett. 2025 May 7;17:249. doi: 10.1007/s40820-025-01760-x (PMC12055708; doi:10.1007/s40820-025-01760-x)
Supplement: Supplementary file 1 — Supplementary file1 (DOCX 10999 KB) [file 40820_2025_1760_MOESM1_ESM.docx]

Supporting Information for

**Indium-MOF as Multifunctional Promoter to Remove Ionic Conductivity and Electrochemical Stability Constraints on Fluoropolymer Electrolytes for All-Solid-State Lithium Metal Battery**

Xiong Xiong Liu^1#^, Long Pan^1#,^*, Haotian Zhang^1^, Cancan Liu^1^, Mufan Cao^1^, Min Gao^1^, Yuan Zhang^1^, Zeyuan Xu^1^, Yaping Wang^1^, ZhengMing Sun^1^*

^1^School of Materials Science and Engineering, Southeast University, Nanjing 211189, P. R. China

^#^ Xiong Xiong Liu and Long Pan contributed equally to this work.

* Corresponding authors. E-mail: [panlong@seu.edu.cn](mailto:panlong@seu.edu.cn) (Long Pan); [zmsun@seu.edu.cn](mailto:zmsun@seu.edu.cn) (ZhengMing Sun)

**S1 Methods**

***Synthesis of In-MOF.*** The synthesis procedure was adopted from previous work [S1]. In brief, 3 g of indium nitrate hydrate (AR, 99.9%, metals basis), 1.661 g of 1,4-dicarboxybenzene (AR, 99%, Macklin), 100 mL of *N*, *N*-dimethylformamide (DMF, AR, 99.5%, Macklin), and 0.5 mL of sodium acetate aqueous solution (0.5M, AR, 99%, Aladdin) were mixed and stirred for 30 min. Then, the solution was heated to 120°C and maintained for two hours. After cooling down to ambient temperature, the white precipitate was washed with anhydrous ethanol three times and dried at 60 °C overnight under vacuum.

***Synthesis of PVH and PVH-IM SPEs.*** They were prepared using a simple solution-cast method, except that the air circulation oven time in this work was set to 3 h. [S2]. Typically, 0.6 g of PVH (average *M*_w_ = 455000, Macklin) and 0.6 g of LiTFSI (99.9%, Macklin) were added in 6 mL of DMF, which was kept stirred until the PVH and LiTFSI were dissolved to form a transparent solution. Subsequently. 0.42 g of In-MOF was added and followed by stirring for another 12 h. The obtained homogeneous dispersion was cast into a petri dish. The petri dish was then directly transferred to an air circulation oven for drying at 30 °C for 3h to remove most of the DMF solvent, followed by drying in a vacuum oven at 120 °C for 24 h.

After the temperature cooled down, a white film was obtained, followed by stored in an Ar-filled glove box for further investigations. The PVH films without In-MOF were prepared by the same procedure without adding In-MOF.

***Materials Characterization.*** A Haoyuan DX-2700BH diffractometer (Cu Kα, λ = 1.5418 Å) was used to record XRD patterns. A FEI Sirion field emission scanning electron microscope was used to study micromorphology. A NETZSCH STA 449F5 thermal gravimetric analyzer was used to record TG curves under nitrogen atmosphere from ambient temperature to 800 °C with a ramping rate of 10 ℃/min. A DSC8000 differential scanning calorimeter was used to record DSC curves under nitrogen atmosphere from −70–200 °C with a ramping rate of 10 ℃/min. A Thermo Scientific Nicolet iS10 infrared spectrometer was used to record FT-IR spectra. A WITec Alpha 300 Access spectrometer was used to record Raman spectra. A Thermo Scientific K-Alpha spectrometer was used to record XPS spectra. A TESCAN BRNO LYRA3 GM focused ion beam-scanning electron microscope was used to record TOF-SIMS spectra.

***Electrochemical tests.*** All cells were assembled using CR2032-type coin cells unless otherwise specified. Symmetric cells with stainless steel (SS) electrodes (*viz*., SS|SPEs|SS) were used to test ionic conductivity. Electrochemical impedance spectroscopy (EIS) profiles were recorded at the frequency range from 10^6^ to 10^−1^ Hz and an applied amplitude of 5 mV. The ionic conductivity was calculated by the following equation:

$$\text{σ}\text{ }\text{= }\frac{\text{L}}{\text{R ×S}}$$

where *R* is the point where the EIS curves intersect the real axis; *L* is the thickness of the solid-state electrolyte; *S* is the cross-sectional area of the solid electrolyte.

The activation energy (*E_a_*) of Li-ion conducting in the SPEs was calculated by the Arrhenius equation:

$$\text{σ}\text{ = }\text{A}\text{e}^{\text{(-}\frac{\text{E}_{\text{a}}}{\text{RT}}\text{)}}$$

where *A* is the preexponential factor, *R* is the universal gas constant, and *T* represents the absolute temperature. Asymmetric SS|SPEs|Li cells were used to test the electrochemical window. LSV curves were recorded at the scanning rate of 0.1 mV s^−1^ from 1.0 to 6.0 V. Li|SPEs|Li symmetric cells were used to test the Li^+^ transference number ($\text{t}_{\text{Li}^{\text{+}}}$) by DC polarization and EIS. The applying voltage of DC polarization is 10 mV, and the EIS profiles were recorded at the frequency range from 10^6^ to 10^−1^ Hz and an applied amplitude of 5 mV. The $\text{t}_{\text{Li}^{\text{+}}}$ was calculated by the following equation:

$$\text{t}_{\text{Li}^{\text{+}}}\text{ }\text{= }\frac{\text{I}_{\text{s}}\text{ (∆V-}\text{I}_{\text{0}}\text{R}_{\text{0}}\text{)}}{\text{I}_{\text{0}}\text{ (∆V-}\text{I}_{\text{s}}\text{R}_{\text{s}}\text{)}}$$

where *I_0_* and *I_s_* stand for the initial and steady-state direct current polarizations, respectively; *∆V* represents the applied polarization voltage (10 mV); *R_0_* and *R_s_* indicate the impedance of Li metal surface passivation layer before and after polarization. The EIS, LSV, and DC polarization were conducted using a BioLogic VMP3 electrochemical station. GCD profiles of symmetric cells were recorded by NEWARE MIHW-200-160CH battery testers at various current densities. All measurements were conducted at 25 °C unless otherwise noted. All cells were rested overnight before testing.

***Full cell assembly.*** LFP was mixed with conductive additive (super P) and binder (PVH-IM) at a weight ratio of 68.3:8.5:23.2. The mixture was dispersed in *N*-methylpyrrolidone (NMP) to form a homogenous slurry, which was then coated on an Al foil, followed by a vacuum-drying at 120 °C for 12 h. The mass loadings of LFP are 1.5–2.5 mg cm^−2^. For the pouch cells, Li foils (50 μm) rolled onto Cu foils were used as anodes. The sizes of LFP cathode and Li anode are 3.4 cm × 4.0 cm and 3.5 cm × 4.5 cm, respectively. The weight of LFP in pouch cells is approximately 33 mg. GCD tests were carried out with a voltage range of 2.8–4.2 V for LFP|Li full cells. All cells were rested overnight before testing. All electrochemical tests were conducted under 25 °C.

***DFT calculations.*** The DFT calculation was performed in periodic boundary conditions utilizing the CP2K/Quickstep package [S3]. The ion-electron interactions were described by the hybrid Gaussian and plane wave (GPW) method [S4]. All elements were optimized in geometric by Gaussian functions which consist of double-ζ plus polarization (DZVP) basis set in a combination of norm conserving Goedecker-Teter-Hutter (GTH) pseudo-potentials [S5-S7]. To avoid the interaction between the adjacent images in Z axis, a vacuum region of more than 15 Å was applied. The exchange-correlation function of Perdew-Burke-Ernzerhof (PBE) was used to optimize the structure under generalized gradient approximation (GGA) **[**S8]. The energy convergent standard was 1 × 10^−6^ Hartree and the energy cutoff was set to be 400 Ry. The structures were fully relaxed until the maximum force on each atom was less than 4.5 × 10^−4^ Hartree/Bohr. The Grimme approach (DFT-D3BJ) was used to conduct vdW correction [S9, S10]. The Multiwfn package was used to process data [S11].

***COMSOL Multiphysics simulations.*** The simulation calculation area is 6 × 6 µm to reveal the growth characteristics of dendrites under different conditions. The simulation time is 150s. The growth behavior of dendrites on the surface of Li metal anodes is simulated by coupling the phase field method, and the governing equations for ion and electron transport [S12-S15]. The interactions between interfacial motions, ion transport, and electric field distributions can be captured by solving these partial differential equations, simulating the growth of dendrites under different conditions.

The phase field equation is as follows:

$$\frac{\text{∂}\text{ξ}}{\text{∂}\text{t}}\text{=}\text{-}\text{L}_{\text{σ}}\left[ \text{g}^{\text{'}}\left( \text{ξ} \right)\text{-}\text{κ}\text{∇}^{\text{2}}\text{ξ} \right]\text{-}\text{L}_{\text{η}}\text{h}^{\text{'}}\left( \text{ξ} \right)\left\{ \exp\left[ \frac{\left( \text{1}\text{-}\text{α} \right)\text{nF}\text{η}_{\text{a}}}{\text{RT}} \right]\text{-}{\tilde{\text{C}}}_{\text{+}}\exp\left[ \frac{\text{-}\text{αnF}\text{η}_{\text{a}}}{\text{RT}} \right] \right\}$$

where $\text{L}_{\text{σ}}$ is interfacial mobility, $\text{L}_{\text{η}}$ is reaction-related constant, $\text{g}\text{(}\text{ξ}\text{)}$ is an arbitrary double well function defined as $\text{g}\text{(}\text{ξ}\text{)}\text{=}\text{W}\text{ξ}^{\text{2}}{\text{(1}\text{-}\text{ξ}\text{)}}^{\text{2}}$ to describe the two equilibrium states for the electrode $\left( \text{ξ}\text{=1} \right)$ and the electrolyte $\left( \text{ξ}\text{=0} \right)$ respectively, W is the barrier height, and $\kappa$ is the gradient coefficient.

$$\text{κ}\text{(}\text{θ}\text{)=}\text{κ}_{\text{0}}\text{[1+}\text{δ}\text{cos(}\text{ωθ}\text{)]}$$

where *δ* and *ω* are the strength and mode of the anisotropy, $\text{κ}_{\text{0}}$ is related to the surface energy, $\text{θ}$ is the angle between the normal vector of interface and the reference axis. $\text{h}\text{(}\text{ξ})$ is an interpolating function defined as $\text{h}\text{(}\text{ξ}\text{)}\text{=}\text{ξ}^{\text{3}}\text{(10}\text{-}\text{15}\text{ξ}\text{+6}\text{ξ}^{\text{2}}\text{)}$. $\eta_{\text{a}}$is the activation overpotential defined as $\text{η}_{\text{a}}\text{=∆}\text{ϕ}\text{-}\text{E}^{\text{θ}}$, where $\text{E}^{\text{θ}}$ is the standard half-cell potential and $\text{∆}\text{ϕ}$ is the overpotential applied in this simulation.${\tilde{\text{C}}}_{\text{+}}\text{ }$is the set of dimensionless concentrations defined as ${\tilde{\text{C}}}_{\text{+}}\text{=}\frac{\text{C}_{\text{Li}^{\text{+}}}}{\text{C}_{\text{0}}}$.

The governing equation for ion transport is as follows:

$$\frac{\text{∂}{\tilde{\text{C}}}_{\text{+}}}{\text{∂}\text{t}}\text{=}\text{∇⋅}\left[ \text{D}^{\text{eff}}\text{∇}{\tilde{\text{C}}}_{\text{+}}\text{+}\frac{\text{D}^{\text{eff}}{\tilde{\text{C}}}_{\text{+}}\text{zF}\text{∇}\text{ϕ}}{\text{RT}} \right]\text{+}\frac{\text{c}_{\text{s}}}{\text{c}_{\text{0}}}\frac{\text{∂}\text{ξ}}{\text{∂}\text{t}}$$

where the $\text{∇⋅}\left[ \text{D}^{\text{eff}}\text{∇}{\tilde{\text{C}}}_{\text{+}} \right]$ describes concentration changes due to diffusion of Li^+^ in the electrolyte.$\text{ }\text{D}^{\text{eff}}$ is effective diffusivity defined as $\text{D}^{\text{eff}}\left( \text{ξ} \right)\text{=}\left( \text{D}_{\text{e}}\text{-}\text{D}_{\text{s}} \right)\text{h}\left( \text{ξ} \right)\text{+}\text{D}_{\text{s}}$, where $\text{D}_{\text{s}}$ and $\text{D}_{\text{e}}$ are the electrode and electrolyte diffusivities respectively. $\text{∇⋅}\left[ \frac{\text{D}^{\text{eff}}{\tilde{\text{C}}}_{\text{+}}\text{zF}\text{∇}\text{ϕ}}{\text{RT}} \right]$ describes the effect of electric fields on the migration of Li^+^, where $\text{z}\text{=1}$, representing the charge carried by a single Li^+^, and $\text{∇}\text{ϕ}$ is the potential gradient. $\frac{\text{C}_{\text{s}}}{\text{c}_{\text{0}}}\frac{\text{∂}\text{ξ}}{\text{∂}\text{t}}$ describes the variation of the Li^+^ concentration during the phase transition caused by the order parameter $\text{ξ}$. $\text{h}\text{(}\xi)$ is used to describe the effect of the variation of the order parameter $\text{ξ}$ on the diffusion coefficient, which makes the effective diffusion coefficient smooth transition in the phase transition region.

The governing equation for electron transport is as follows:

$$\text{∇⋅}\left[ \text{σ}\left( \text{ξ} \right)\text{∇}\text{ϕ}\left( \text{r}\text{,}\text{t} \right) \right]\text{=}\text{I}_{\text{R}}\text{=}\text{nF}\text{c}_{\text{s}}\frac{\text{∂}\text{ξ}}{\text{∂}\text{t}}$$

where is$\text{ }\text{σ}\left( \text{ξ} \right)$ is effective conductivity defined as $\sigma\text{(}\xi)\text{=}\left( \text{σ}_{\text{e}}\text{-}\text{σ}_{\text{s}} \right)\text{h}\left( \text{ξ} \right)\text{+}\text{σ}_{\text{s}}$, describing how the conductivity varies with the sequence parameter $\text{ξ}$. $\text{Σ}_{\text{e}}$ and $\text{σ}_{\text{s}}$ are the electronic conductivity in the electrode and electrolyte, respectively. $\text{h}\text{(}\text{ξ})$ allows a smooth transition of the conductivity change in the phase transition region.

**Supplementary Figures**


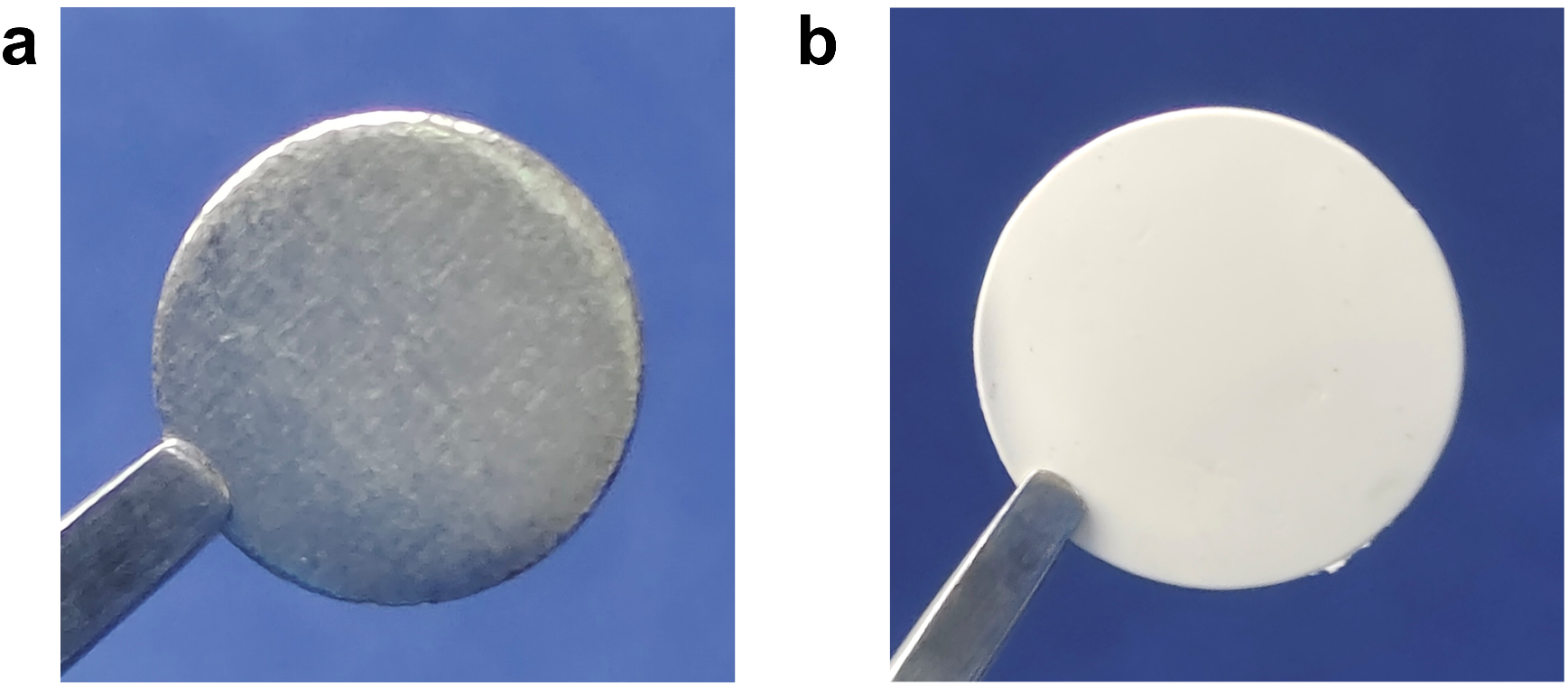


**Fig. S1** Photographs of (**a**) fresh Li metal and (**b**) as-prepared PVH film


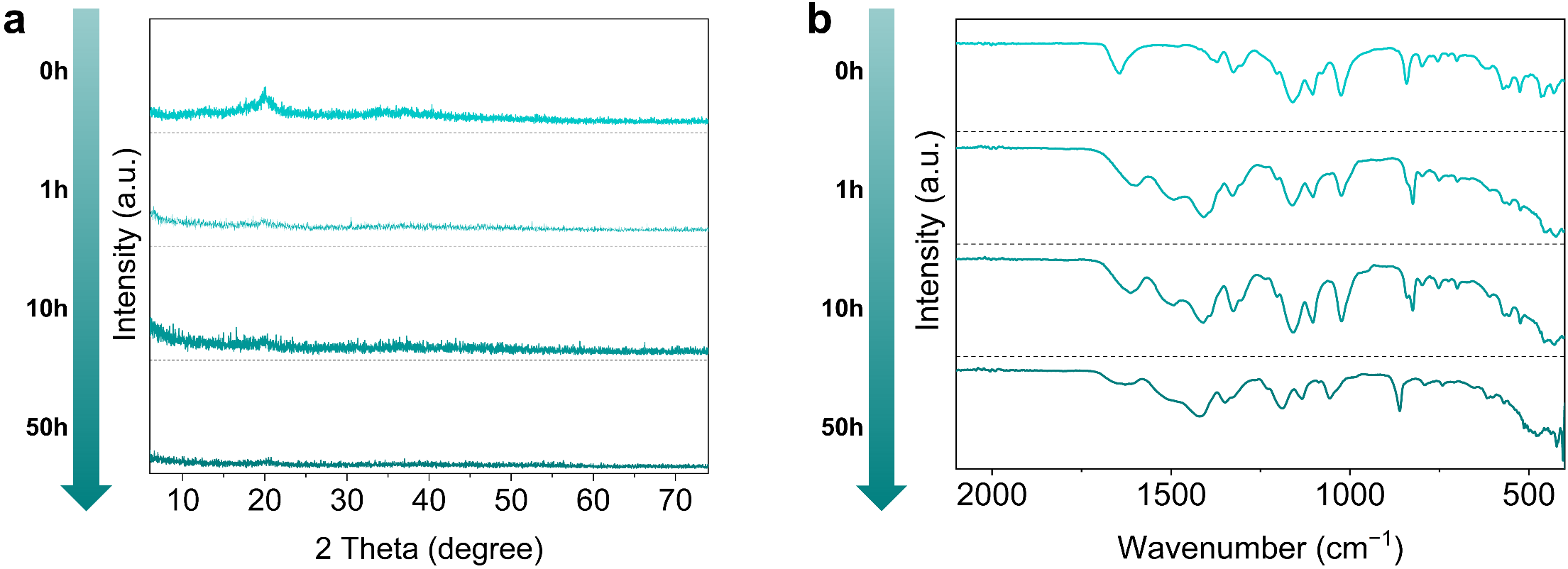


**Fig. S2** (**a**) XRD pattern and (**b**) FT-IR spectra of PVH after 0, 1, 10, and 50 h cycling at 0.1 mA cm^−2^


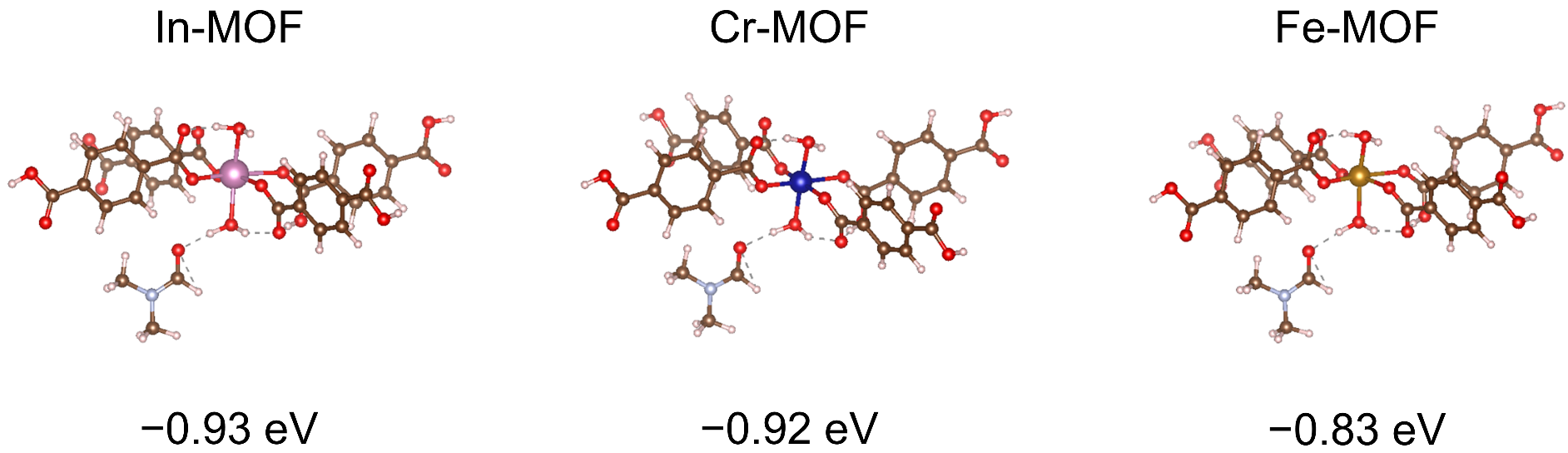


**Fig. S3** Adsorption energies of In-MOF/DMF, Cr-MOF/DMF, and Fe-MOF/DMF


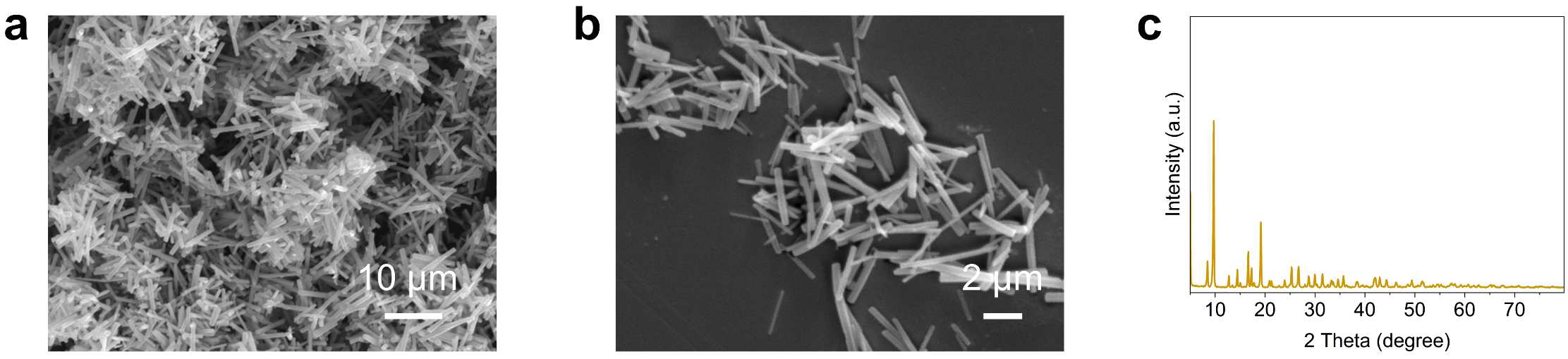


**Fig. S4** (**a, b**) SEM images and (**c**) XRD pattern of 1D In-MOF nanorods


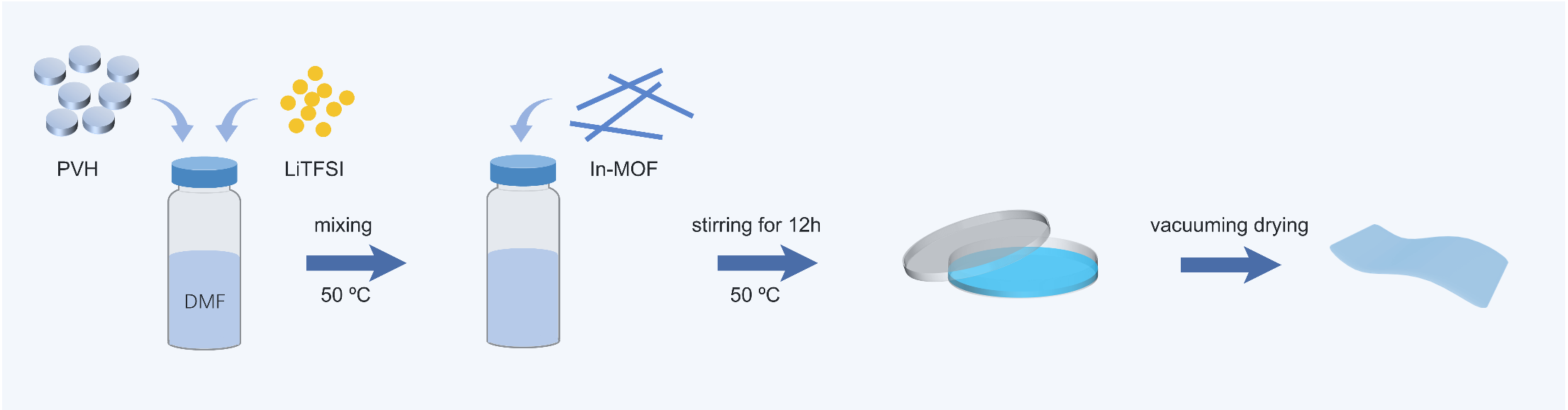


**Fig. S5** Schematic of the preparation process of PVH-IM

**
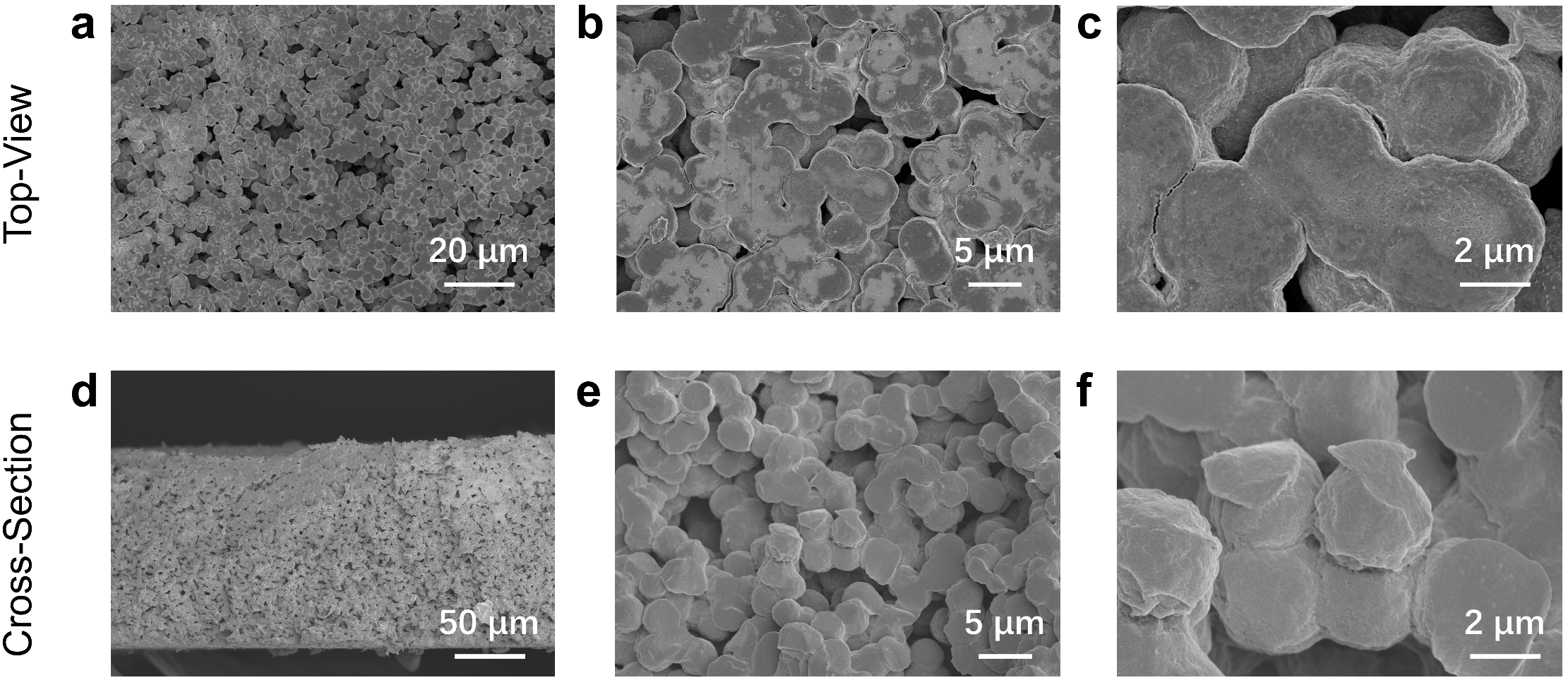
**

**Fig. S6 (a–c)** Top-view and **(c–e)** cross-sectional SEM images of PVH

**
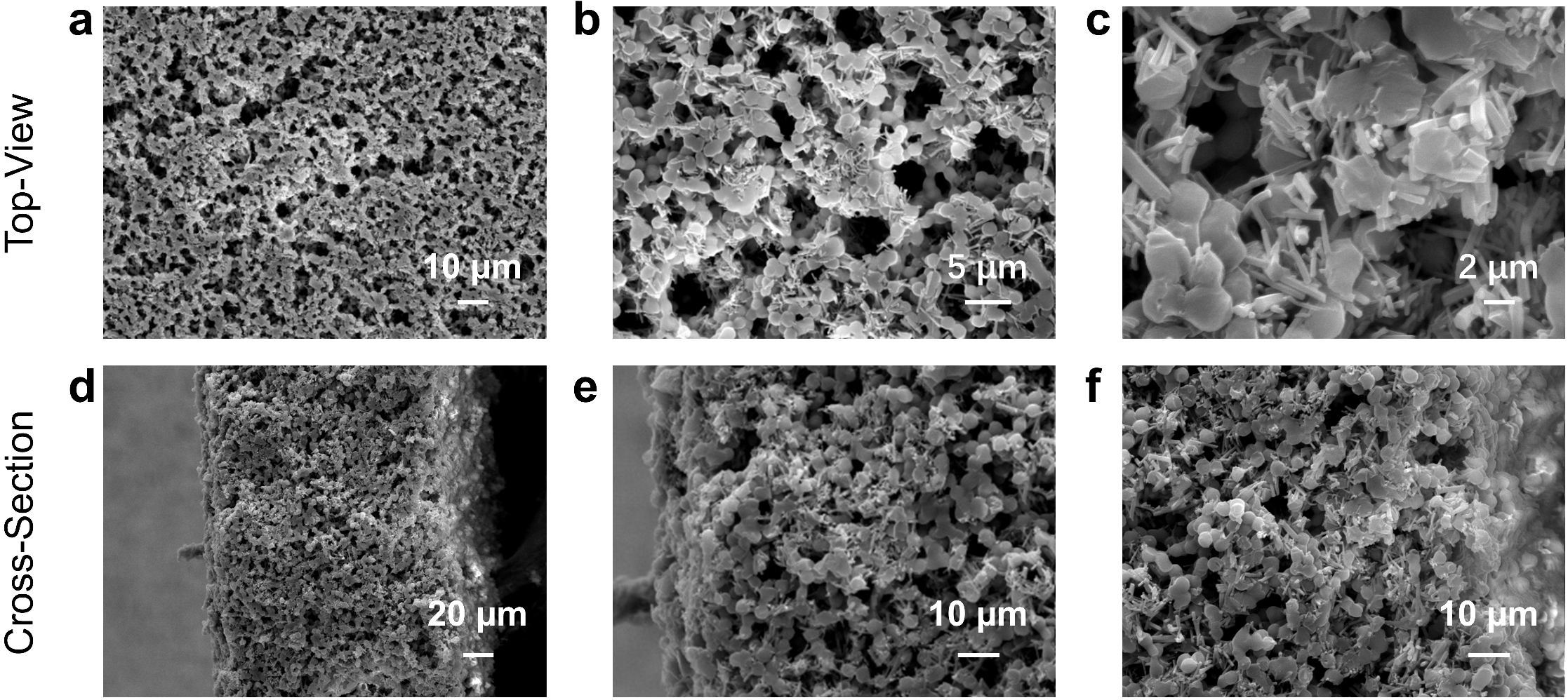
**

**Fig. S7 (a–c)** Top-view and **(c–e)** cross-sectional SEM images of PVH-IM. Note that the In-MOF nanorods dispersed uniformly in both lateral and vertical directions, as reflected in the top-view and cross-sectional SEM images, respectively


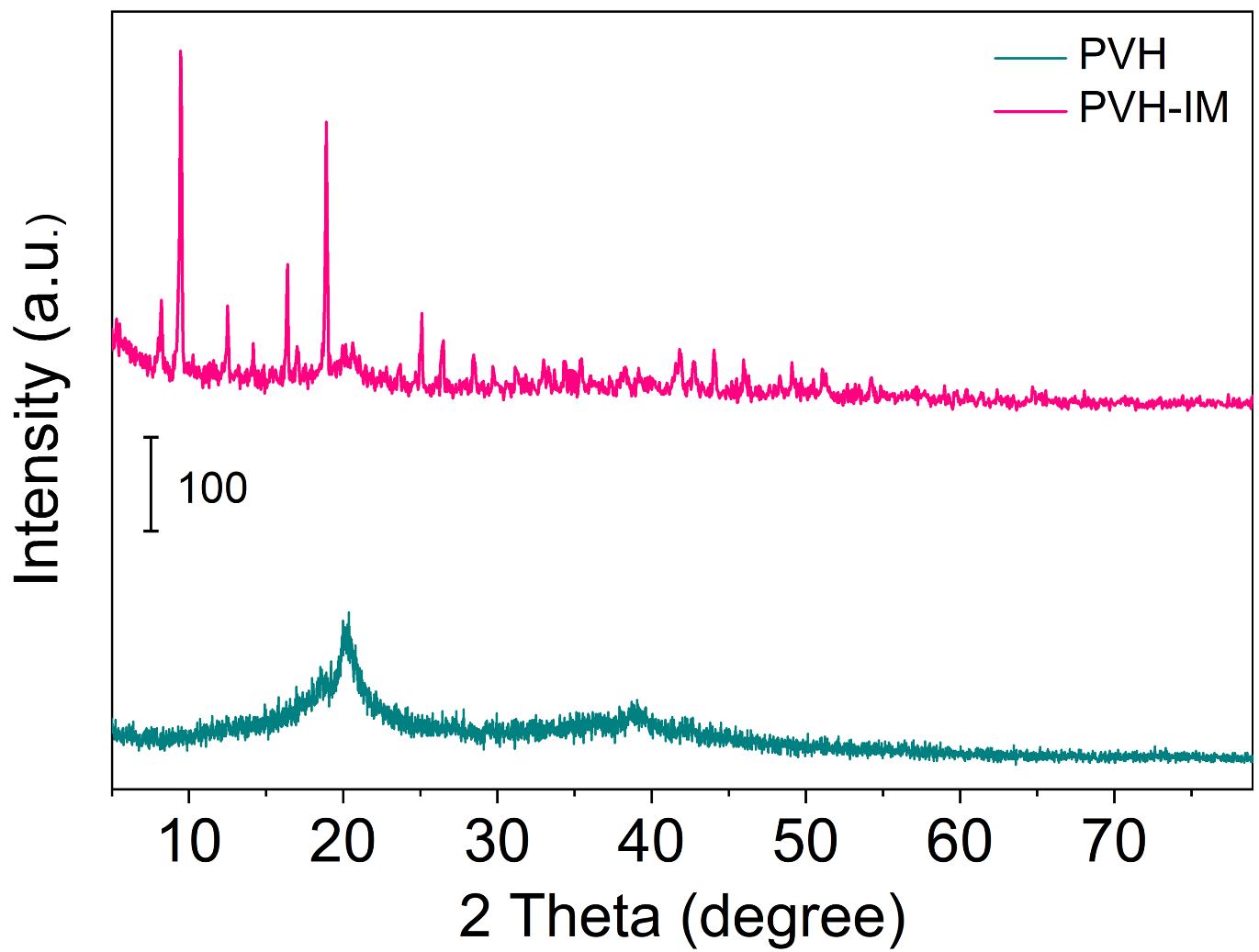


**Fig. S8** XRD patterns of PVH and PVH-IM


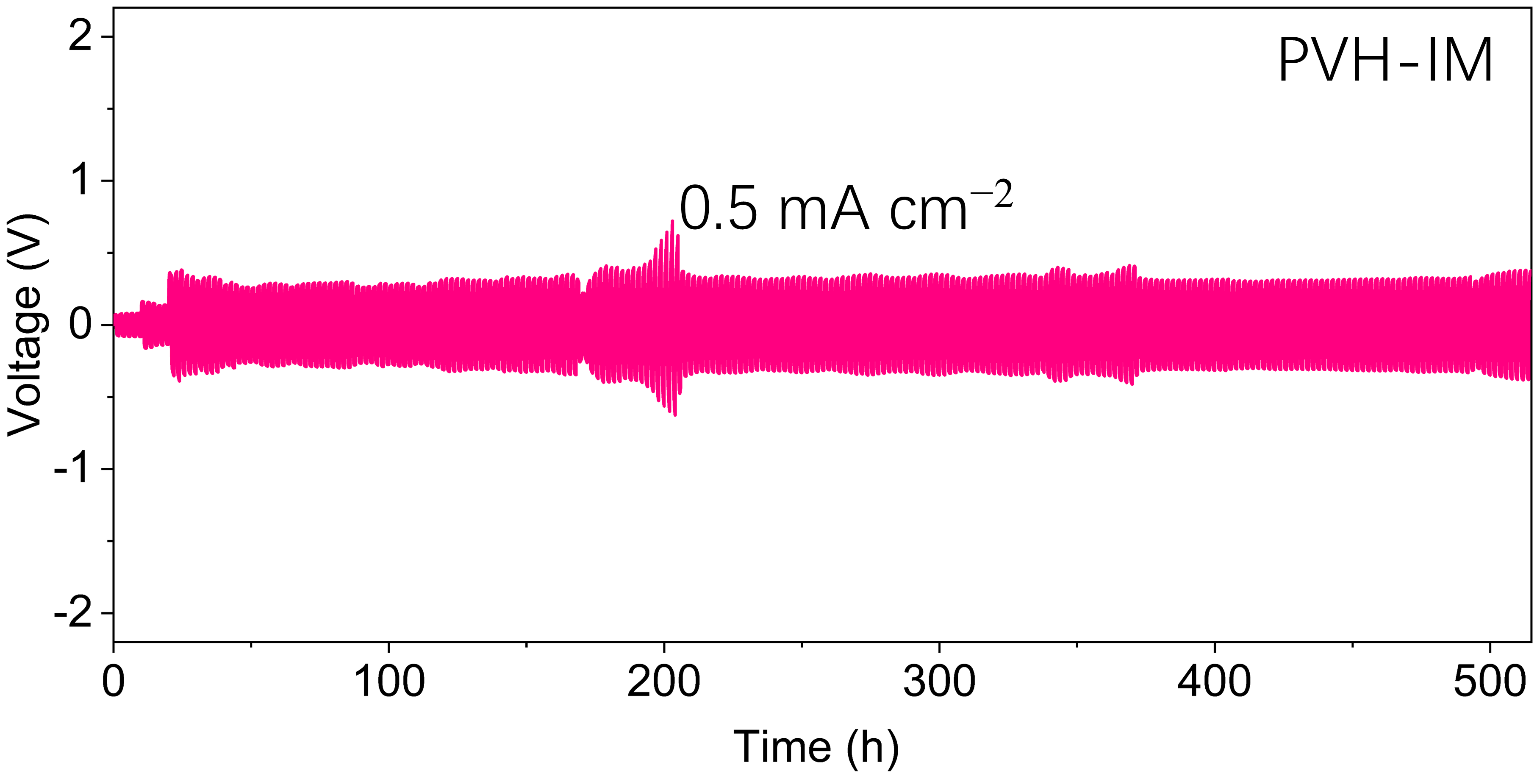


**Fig. S9** Galvanostatic voltage profiles of Li|PVH-IM|Li symmetric cells at 0.5 mA cm^−2^


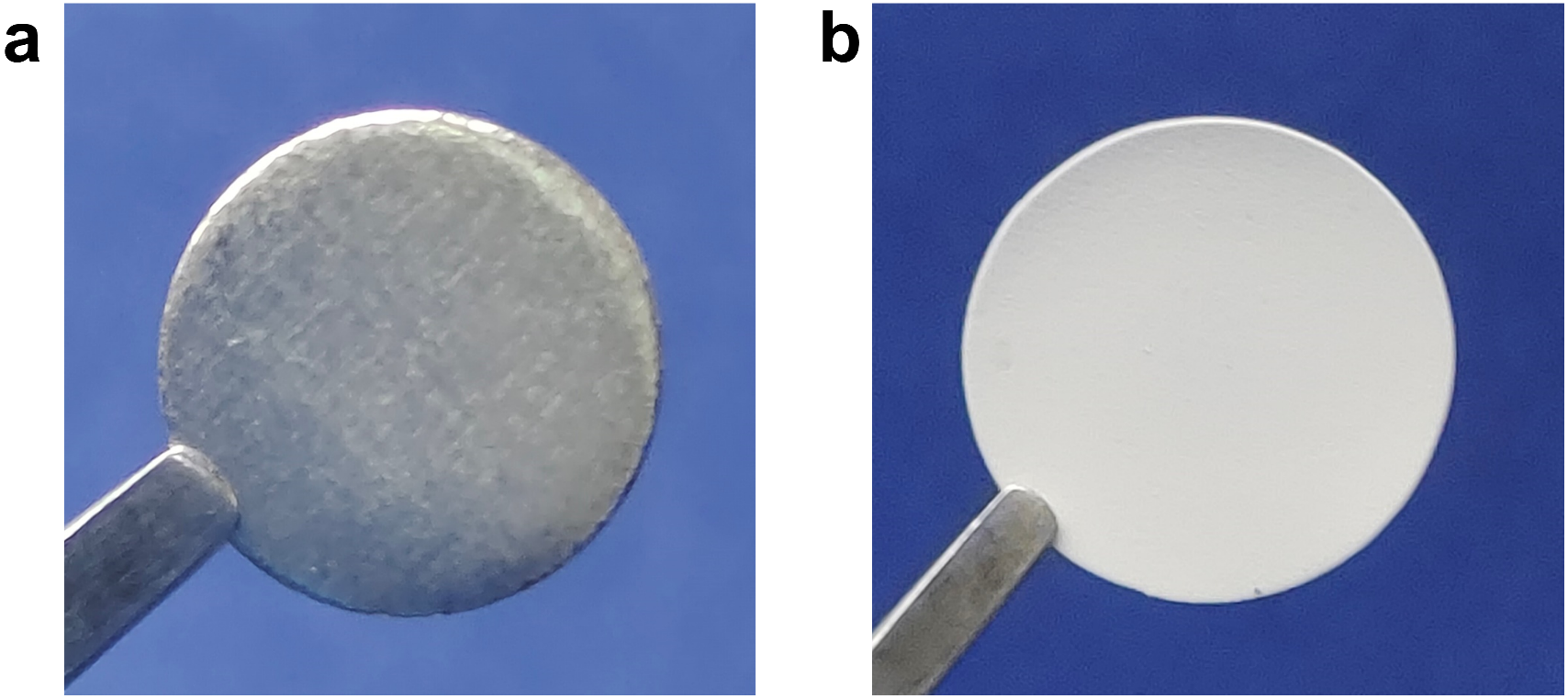


**Fig. S10** Photographs of (**a**) fresh Li metal and (**b**) as-prepared PVH-IM film


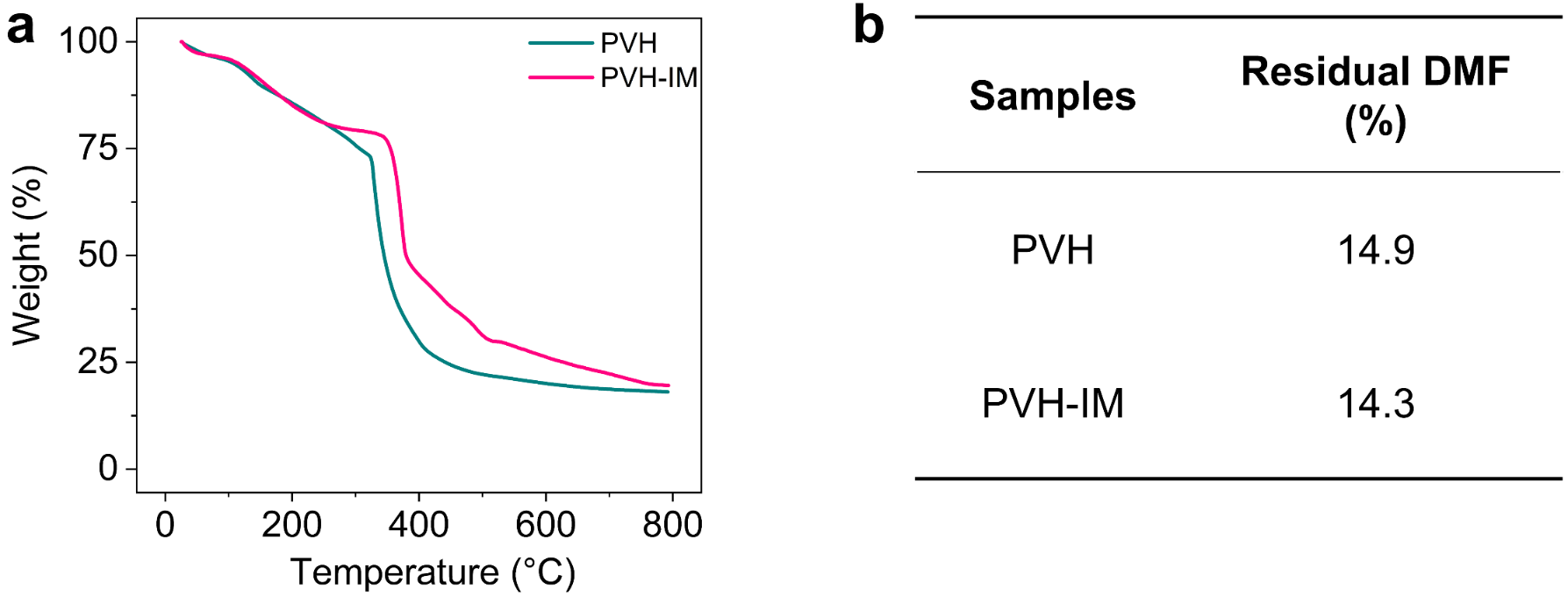


**Fig. S11** (**a**) TG curves and (**b**) corresponding residual DMF contents of PVH and PVH-IM. The residual DMF contents were calculated based on the total weight loss from ambient temperature to 200 °C


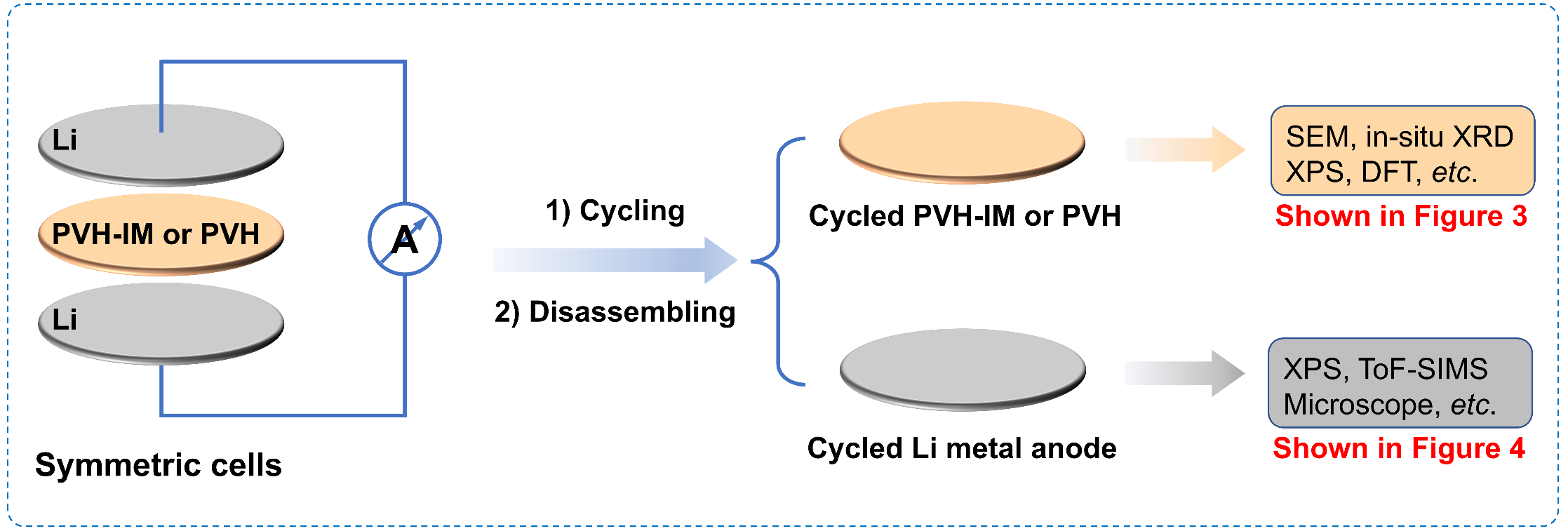


**Fig. S12** Schematic of how to reveal the enhancement mechanism of In-MOF on the PVH's electrochemical stability against Li metal anodes


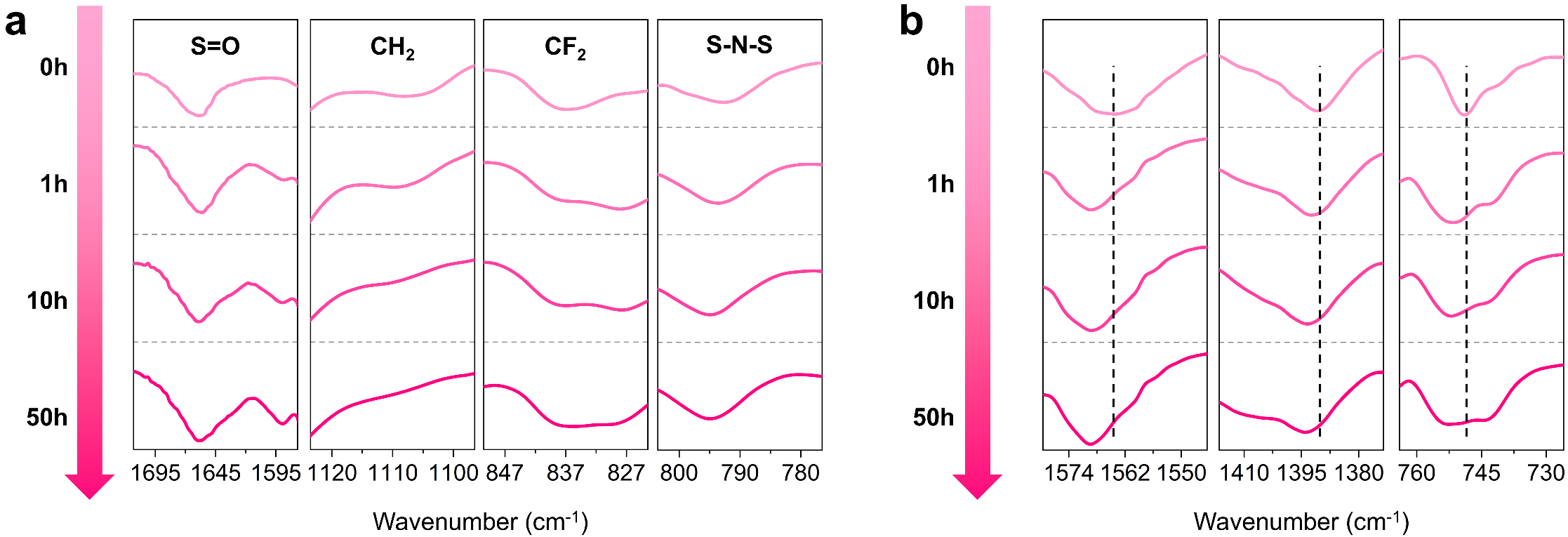


**Fig. S13** FT-IR spectra of PVH-IM after 0, 1, 10, and 50 h cycling at 0.1 mA cm^−2^

The peaks in (a), corresponding to those in **Figure 1d**, are attributed to the PVH matrix. The characteristic peaks of –CF_2_– and –CH_2_– are still maintained, indicating that the limited reaction between PVH and Li metal anodes. The peaks at 1564.5 cm^−1^, 1390.9 cm^−1^, and 749.2 cm^−1^ in (b) are attributed to the In-MOF. These peaks shift to 1568.8 cm^−1^, 1393.3 cm^−1^, and 752.6 cm^−1^, respectively, indicating that the In-MOF first reacts with the Li metal anodes during cycling. The results support that the PVH matrix is protected by In-MOF.


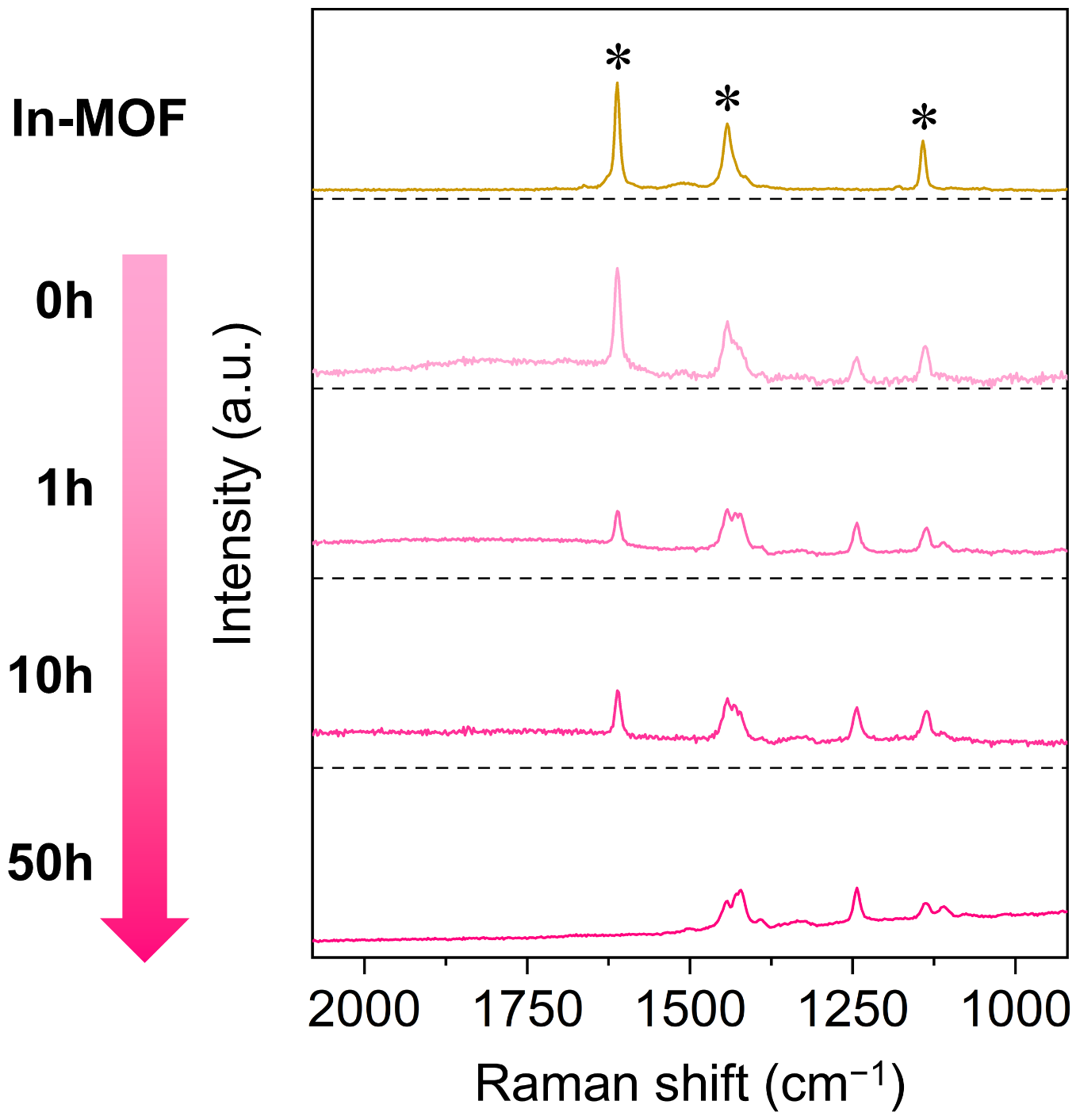


**Fig. S14** Raman spectra of In-MOF and PVH-IM after 0, 1, 10, and 50 h cycling at 0.1 mA cm^−2^.

The peaks at 1444.7, 1421.5, 1391.3, 1243.4, 1138.4, and 1109.5 cm^−1^, corresponding to those in **Figure 1e,** are attributed to PVH matrix. After 50h cycling, these peaks are well maintained, suggesting that weak reaction between PVH and Li metal anodes. Other peaks at 1611.5, 1442.7, and 1141.9 cm^−1^ (marked with star symbol) are attributed to In-MOF. These peaks vanish after 50 h cycling, implying In-MOF reacts with the Li metal anodes during cycling. The Raman results further prove that In-MOF protects PVH from the Li metal anodes.


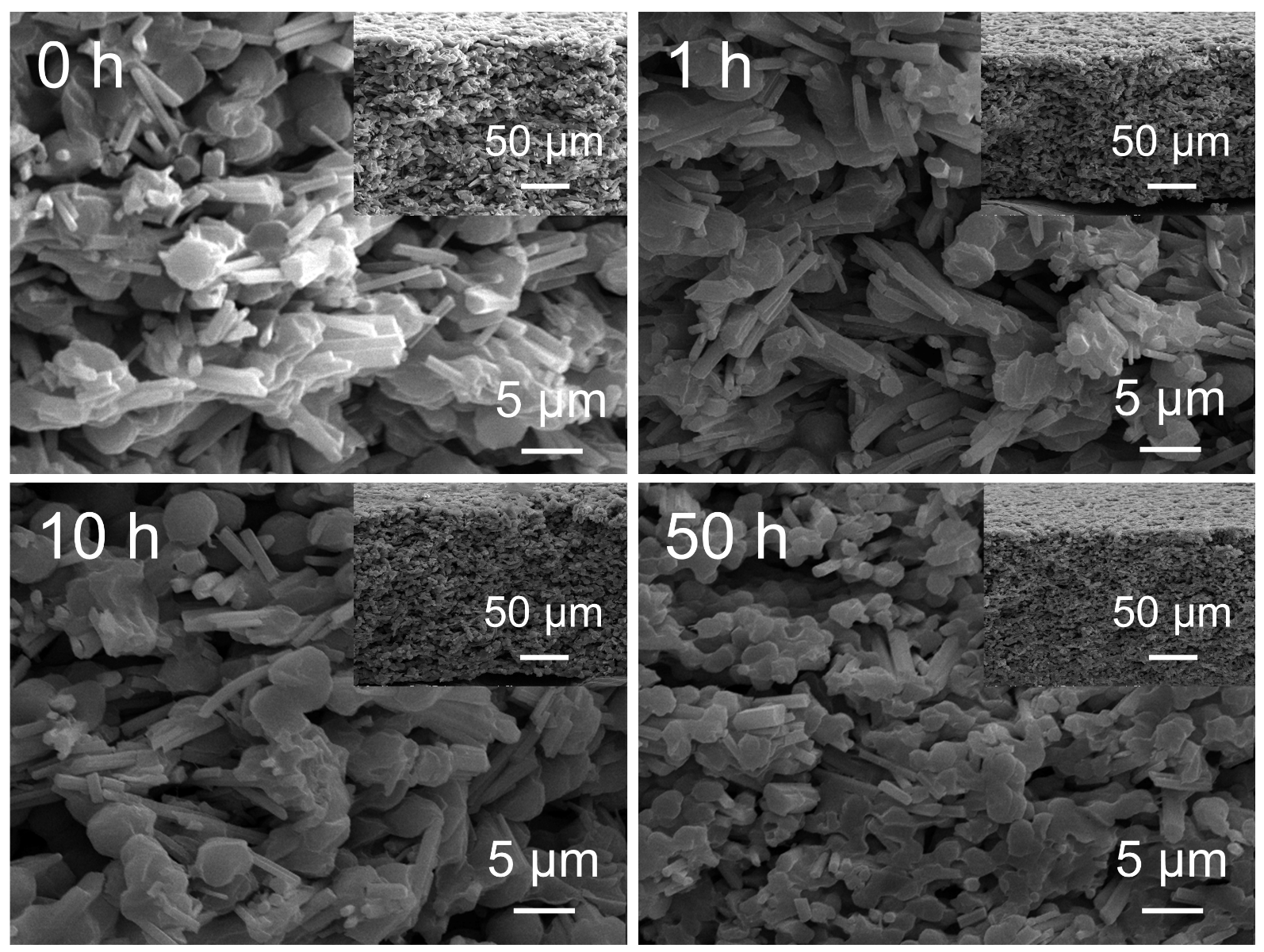


**Fig. S15** Cross-section SEM images of PVH-IM after 0, 1, 10, and 50 h cycling at 0.1 mA cm^−2^


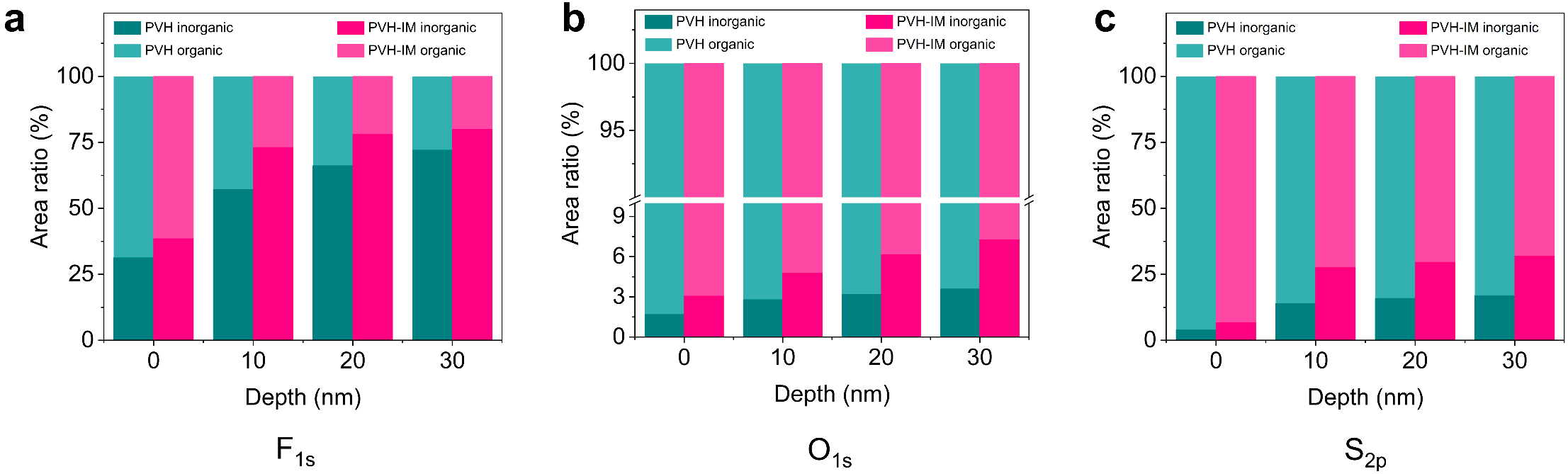


**Fig. S16** The relative content of inorganics and organics components calculated from the (**a**) F 1s, (**b**) O 1s, and (**c**) S 2p spectra of PVH and PVH-IM in **Fig. 4a, b** after 50h cycling at 0.1 mA cm^−2^


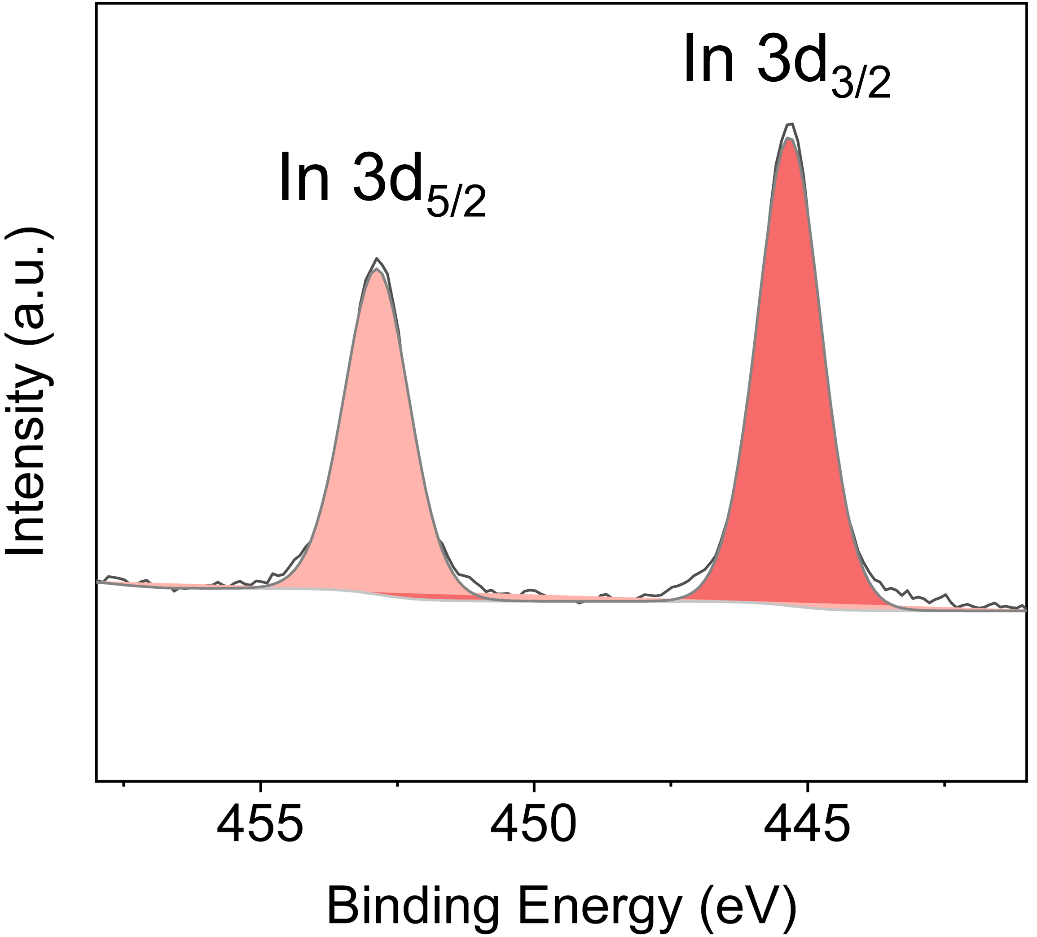


**Fig. S17** In 3d XPS spectrum of PVH-IM


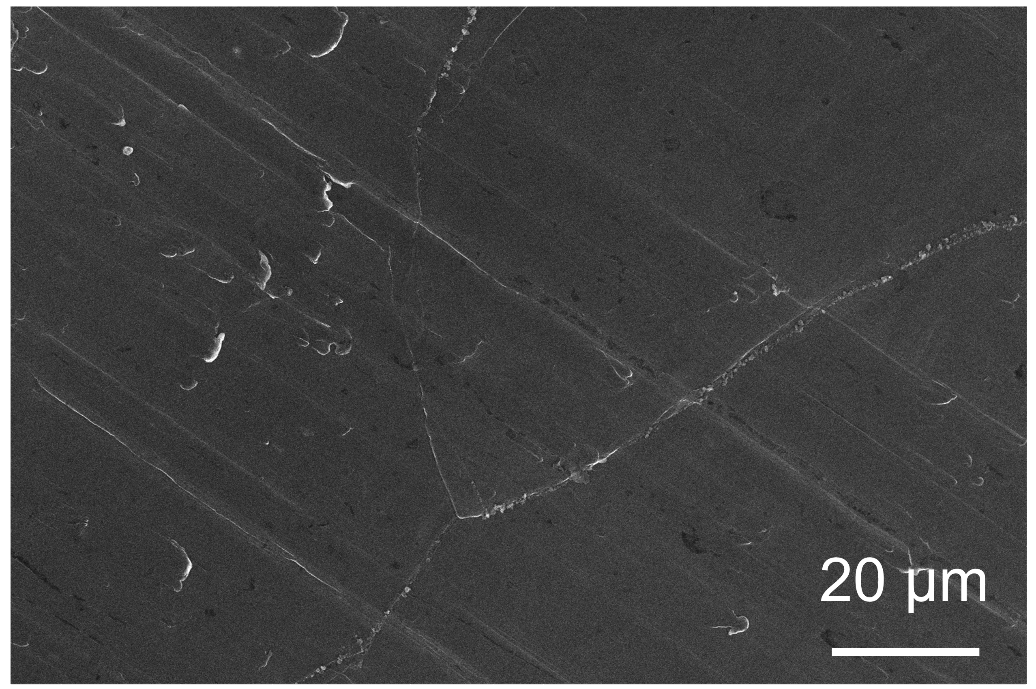


**Fig. S18** SEM image of fresh Li metal surface


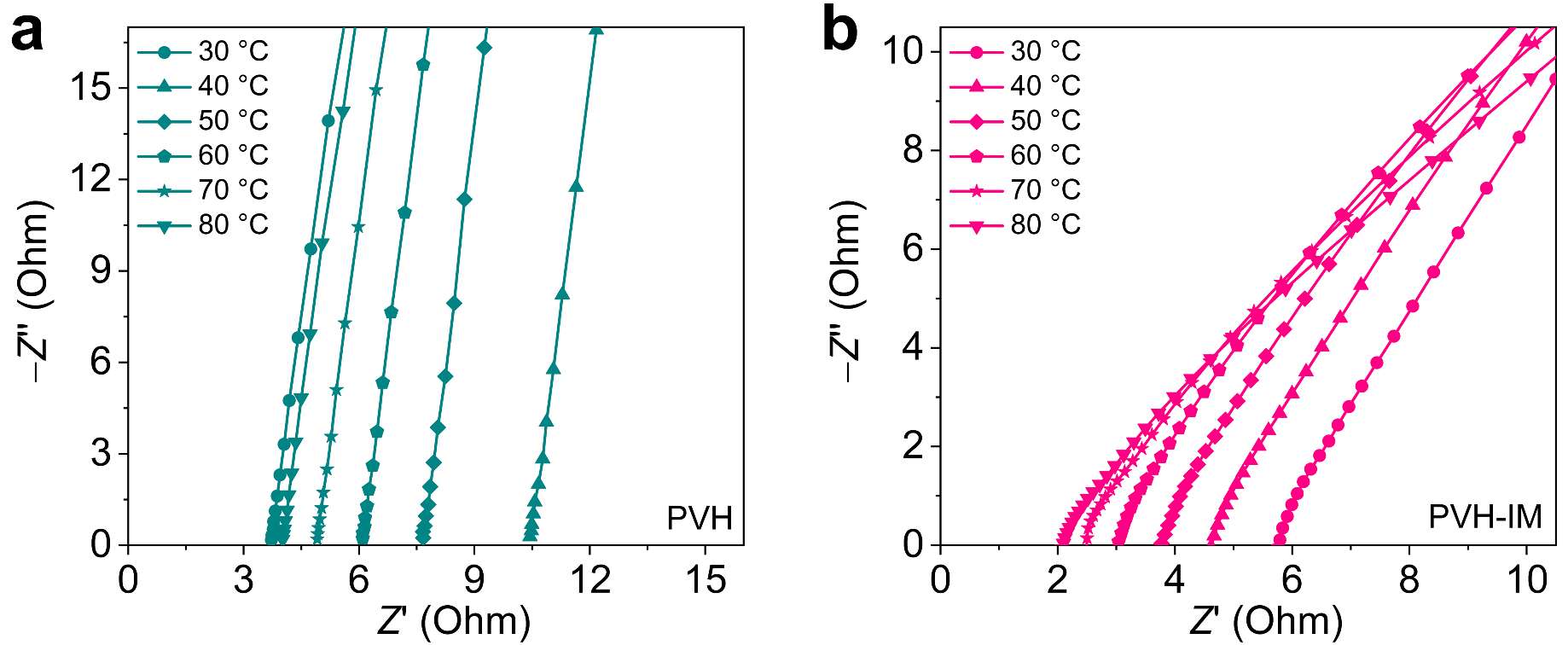


**Fig. S19** EIS curves of (**a**) SS|PVH|SS and (**b**) SS|PVH-IM|SS symmetric cells at different temperatures


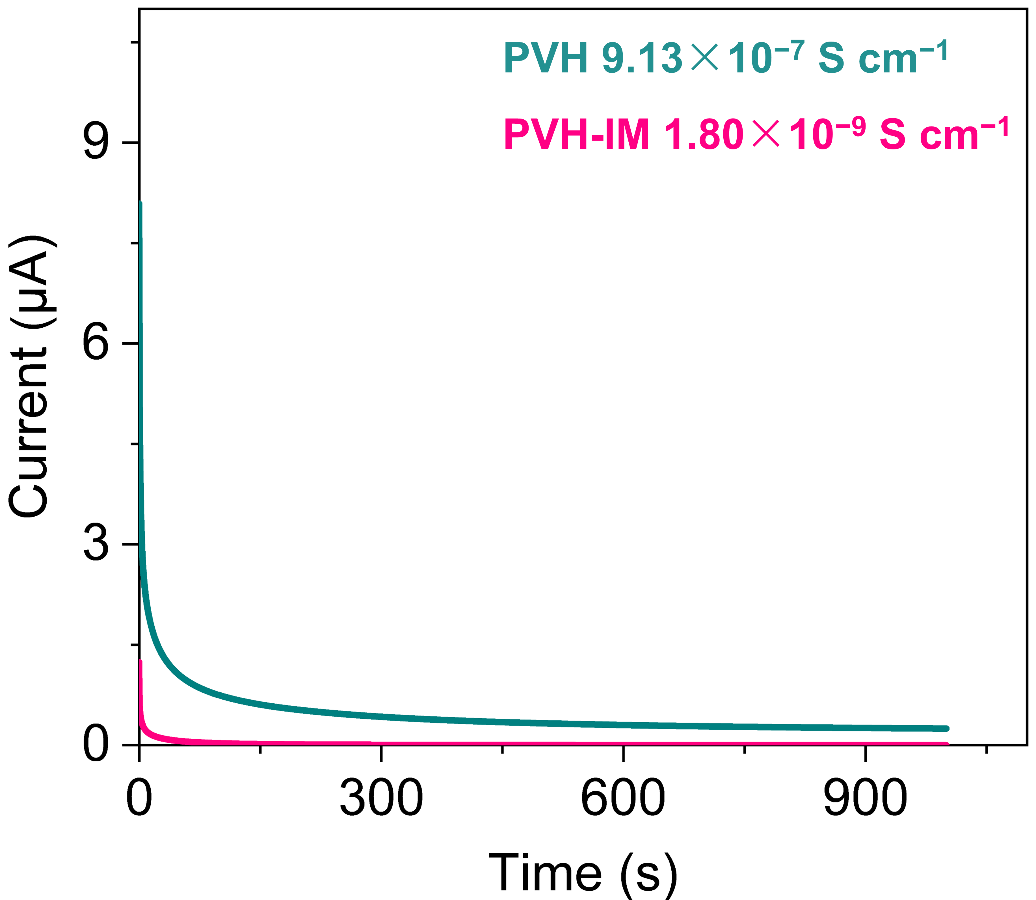


**Fig. S20** Electronic conductivities of PVH and PVH-IM


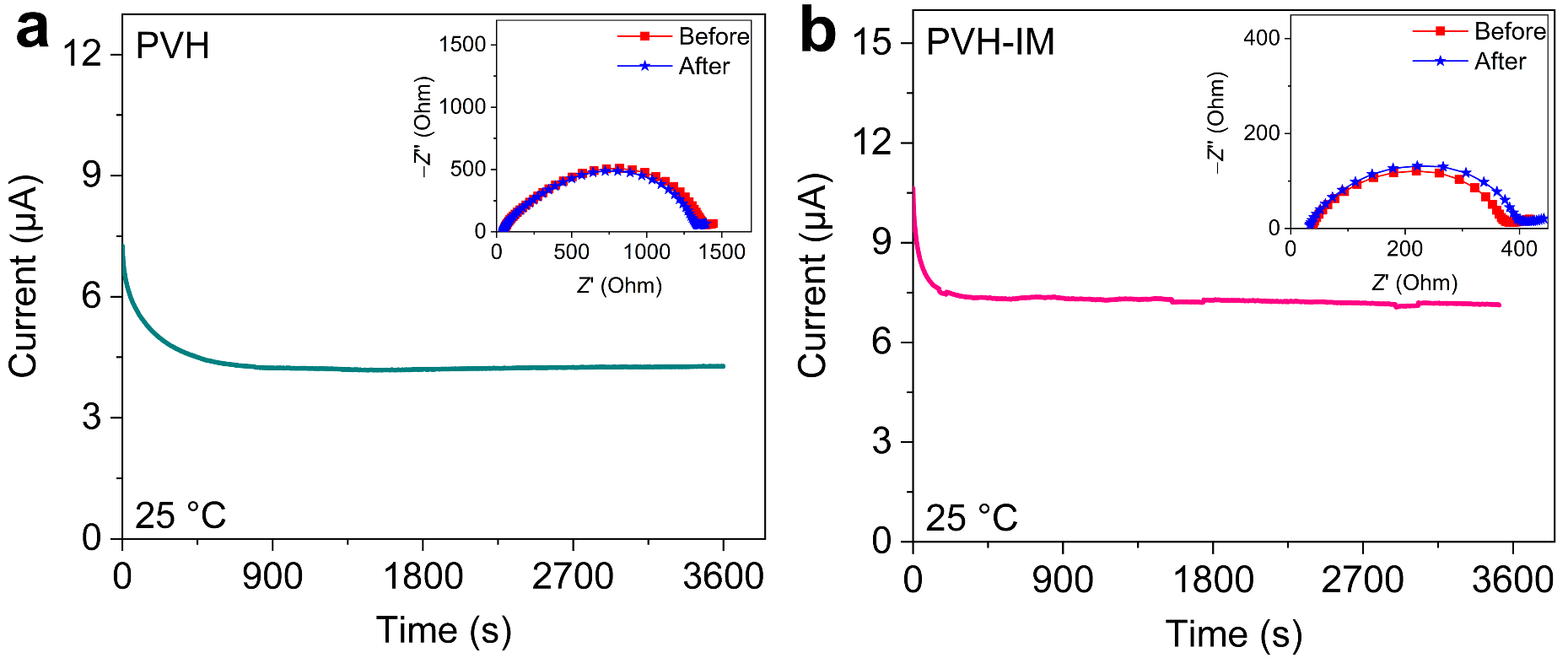


**Fig. S21** Typical DC polarization curves of (**a**) Li|PVH|Li and (**b**) Li|PVH-IM|Li symmetric cells. The insets are the corresponding EIS curves before and after the DC polarization. All measurements were conducted with a DC polarization voltage of 10 mV and at 25 °C


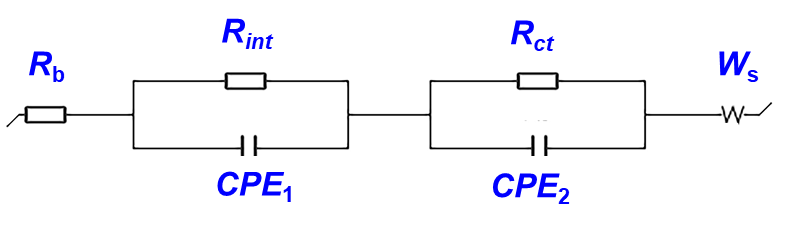


The EIS profiles before and after polarization were fitted using the following equivalent circuit. R_b_ represents the impedance of SPE; R_int_ denotes the interfaces impedance between the PVH and In-MOF, and R_ct_ represents the impedance of charge transfer at the SPE (*i*.*e*., PVH and PVH-IM) and electrode (*i*.*e*., Li metal surface) interfaces. R_ct_ truly reflects the impedance changes of the passivation layer on the lithium metal surface. Therefore, R_0_ and R_s_ in the Bruce-Vincent-Evans equation are equal to R_ct_ before and after polarization, respectively.


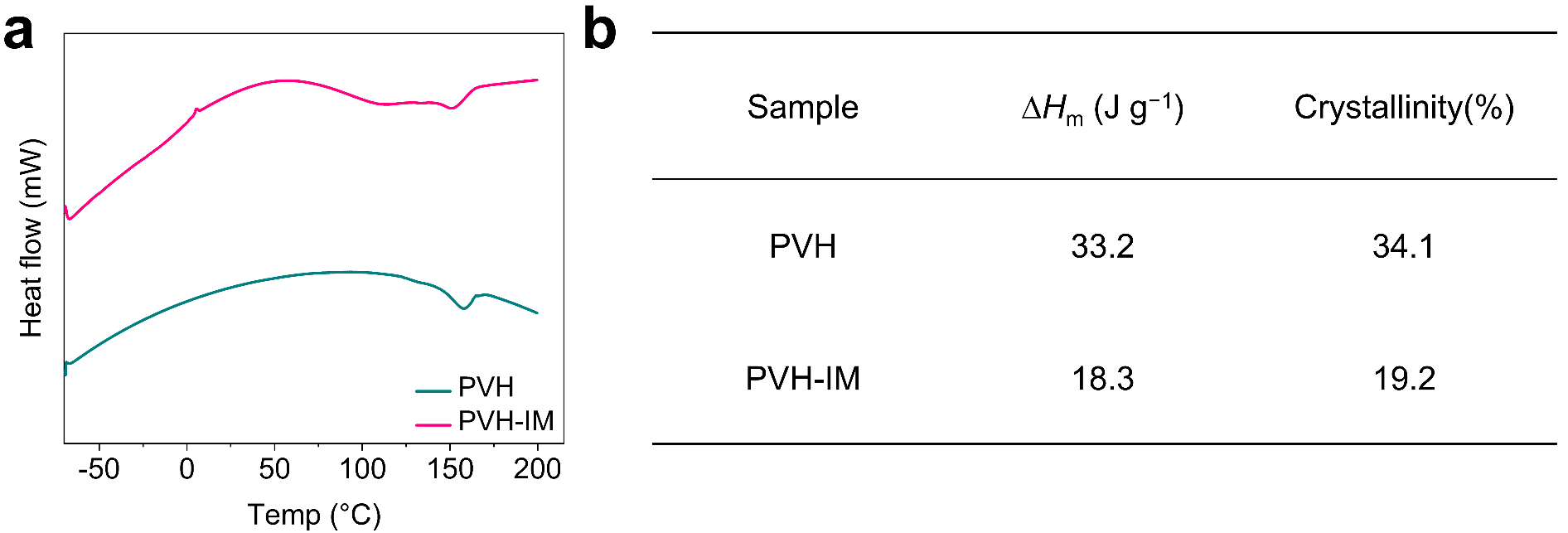


**Fig. S22** (**a**) DSC curves and (**b**) corresponding crystallinity of PVH and PVH-IM

The crystallinity is calculated based on the following equation: $\chi_{c}= \frac{\Delta H_{m}}{{\Delta H}_{m}^{0}} \times100$. ${\Delta H}_{m}^{0}$ is the fusion enthalpy for 100% crystallinity. For PVDF-HFP, the value of 97.5 J g^−1^ is used [S15].


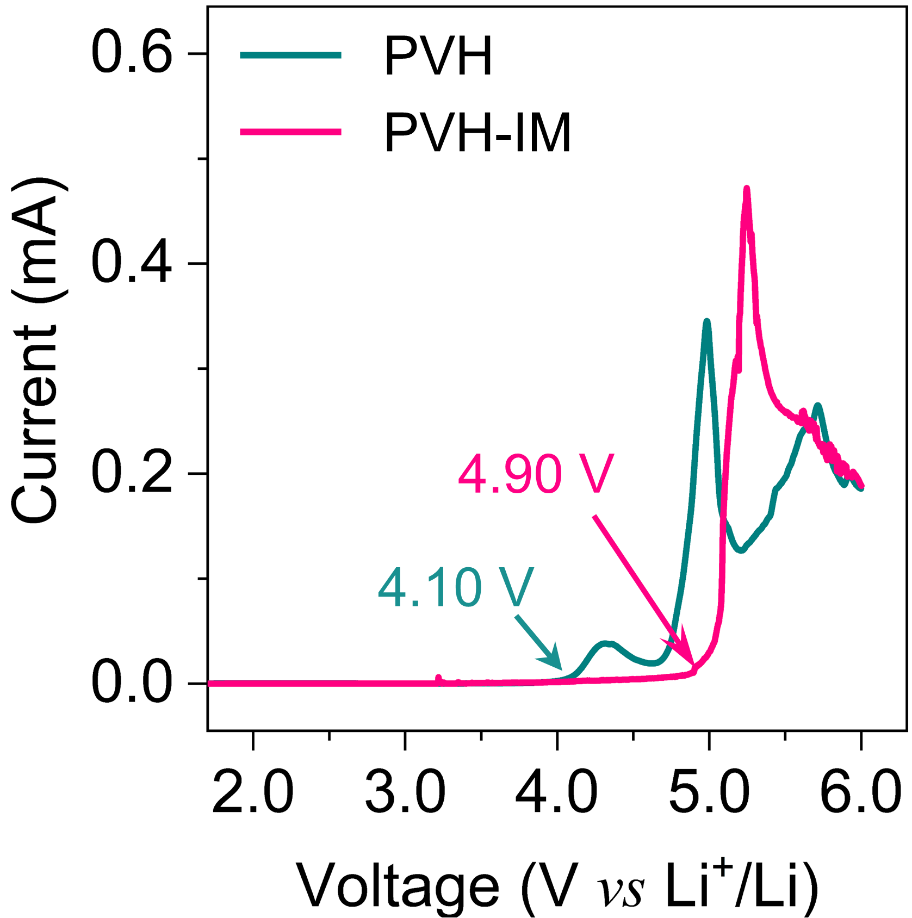


**Fig. S23** LSV curves of PVH and PVH-IM at 25 °C


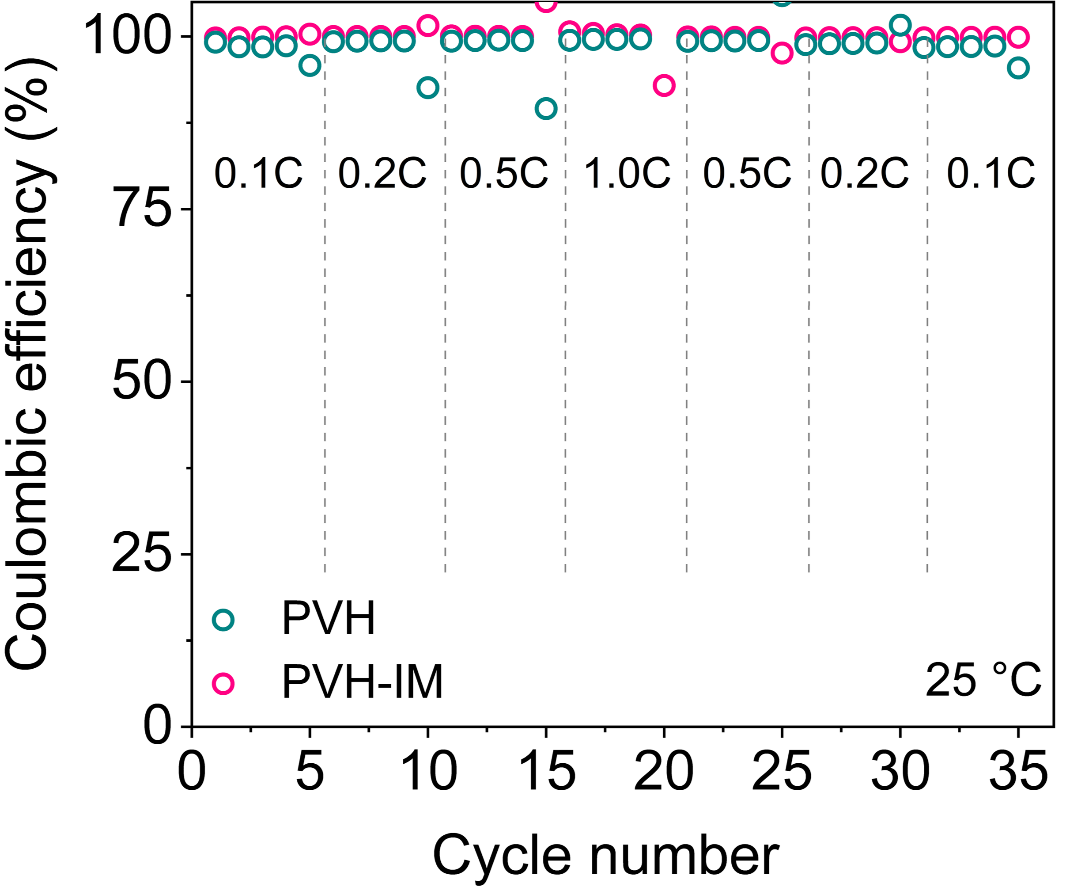


**Fig. S24** Coulombic efficiency of all-solid-state LFP|PVH|Li and LFP|PVH-IM|Li full cells at various current densities under 25 °C


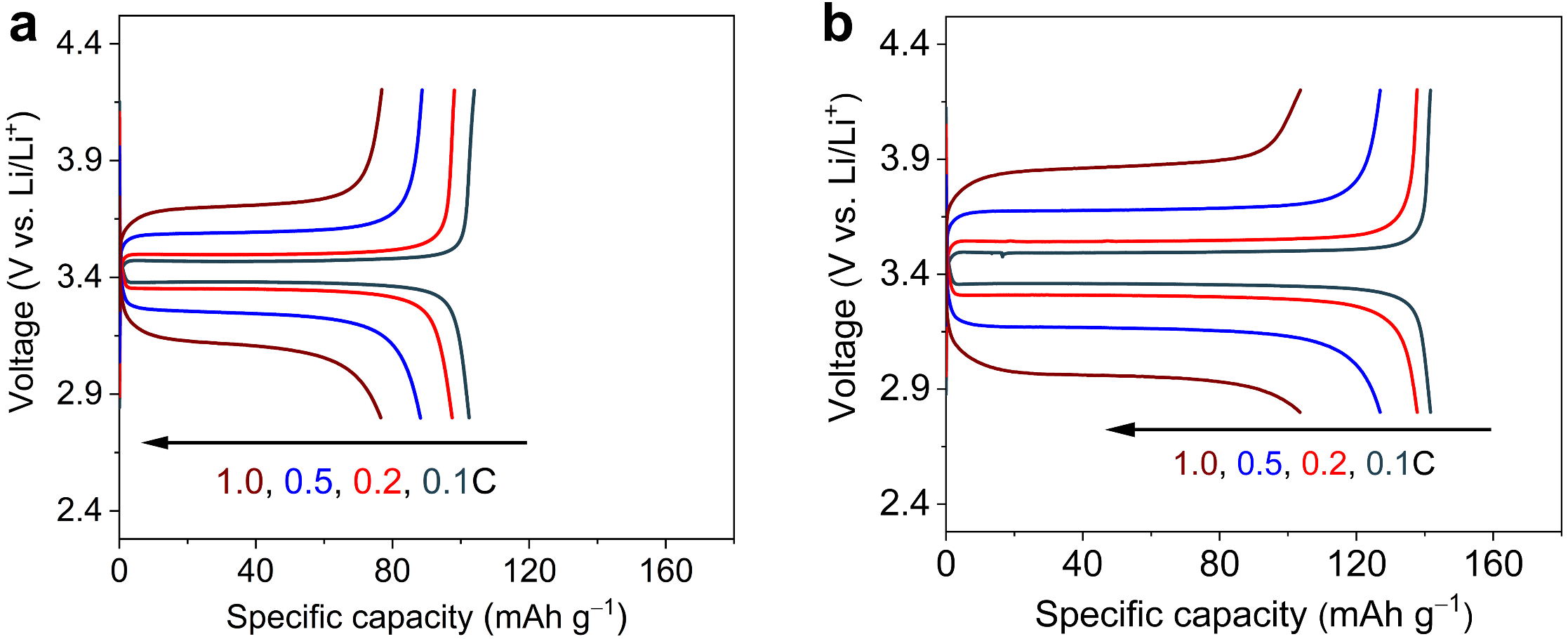


**Fig. S25** GCD curves of (**a**) LFP|PVH|Li and (**b**) LFP|PVH-IM|Li full cells at various current densities under 25 °C


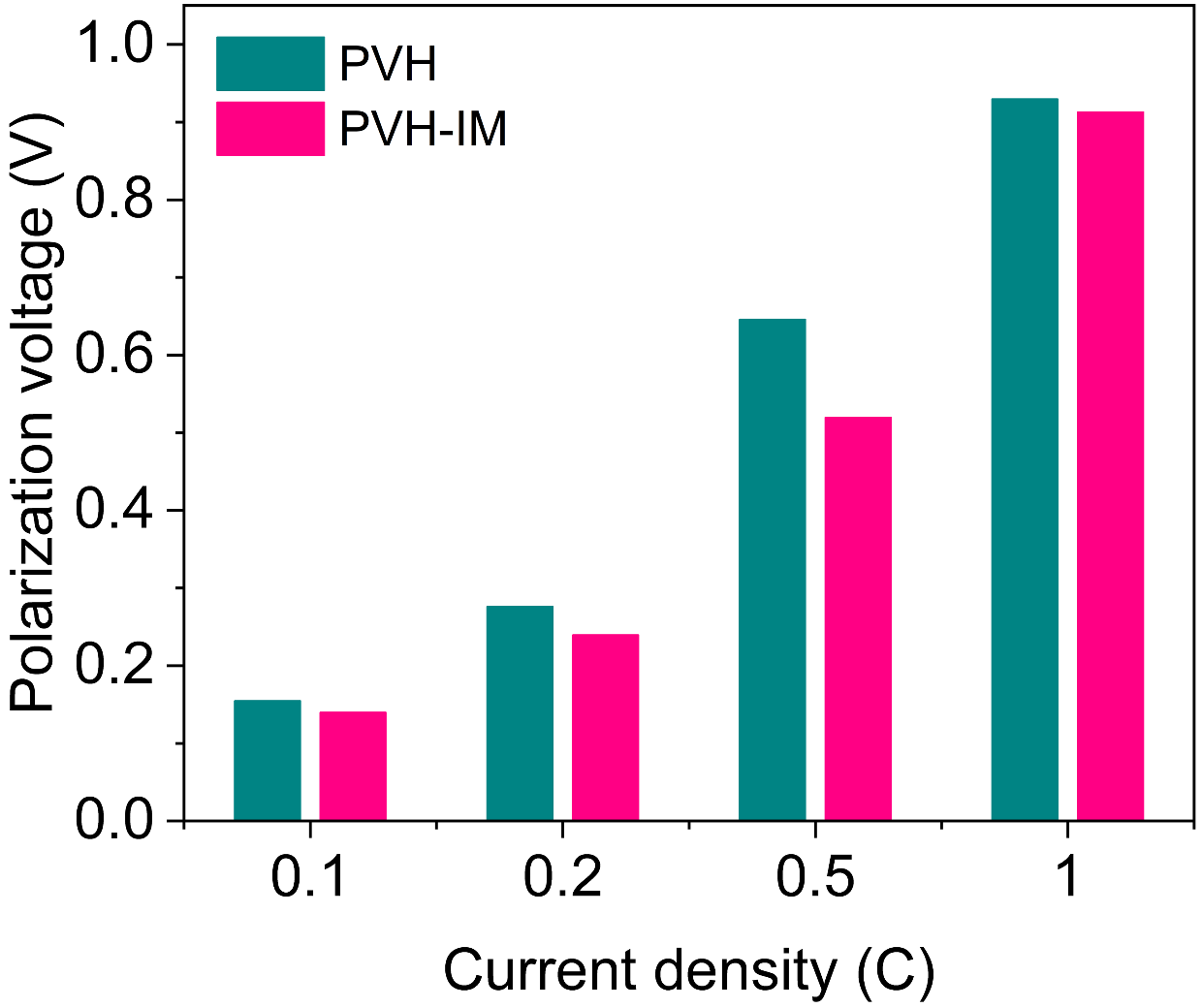


**Fig. S26** Polarization voltages of LFP|PVH|Li and LFP|PVH-IM|Li full cells at various current densities under 25 °C


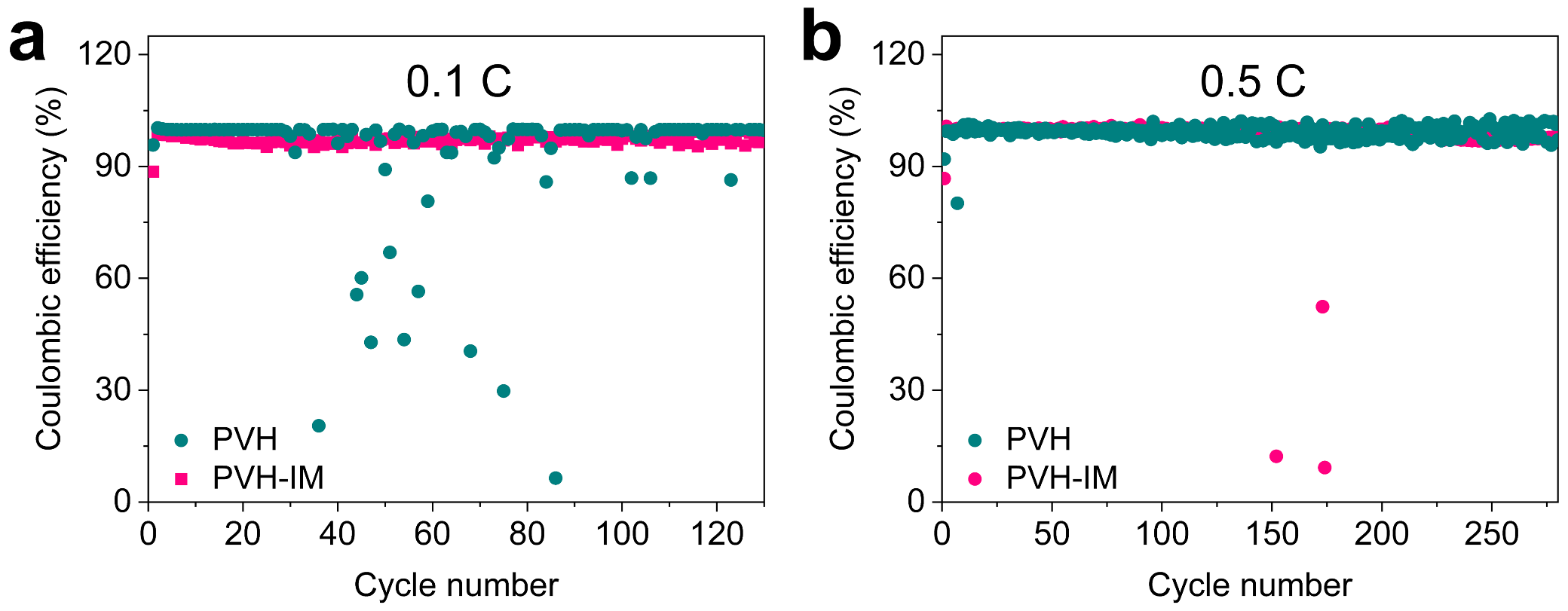


**Fig. S27** Coulombic efficiency at the current density of (**a**) 0.1C and (**b**) 0.5C of all-solid-state LFP|PVH|Li and LFP|PVH-IM|Li full cells


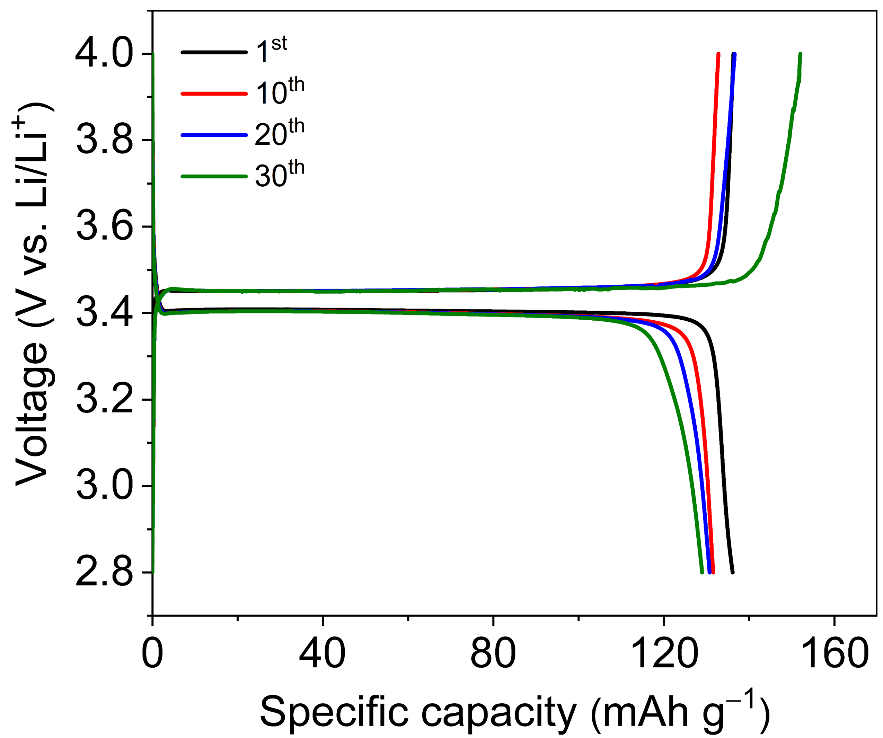


**Fig. S28** GCD curves of LFP|PVH-IM|Li pouch cells under 25 °C


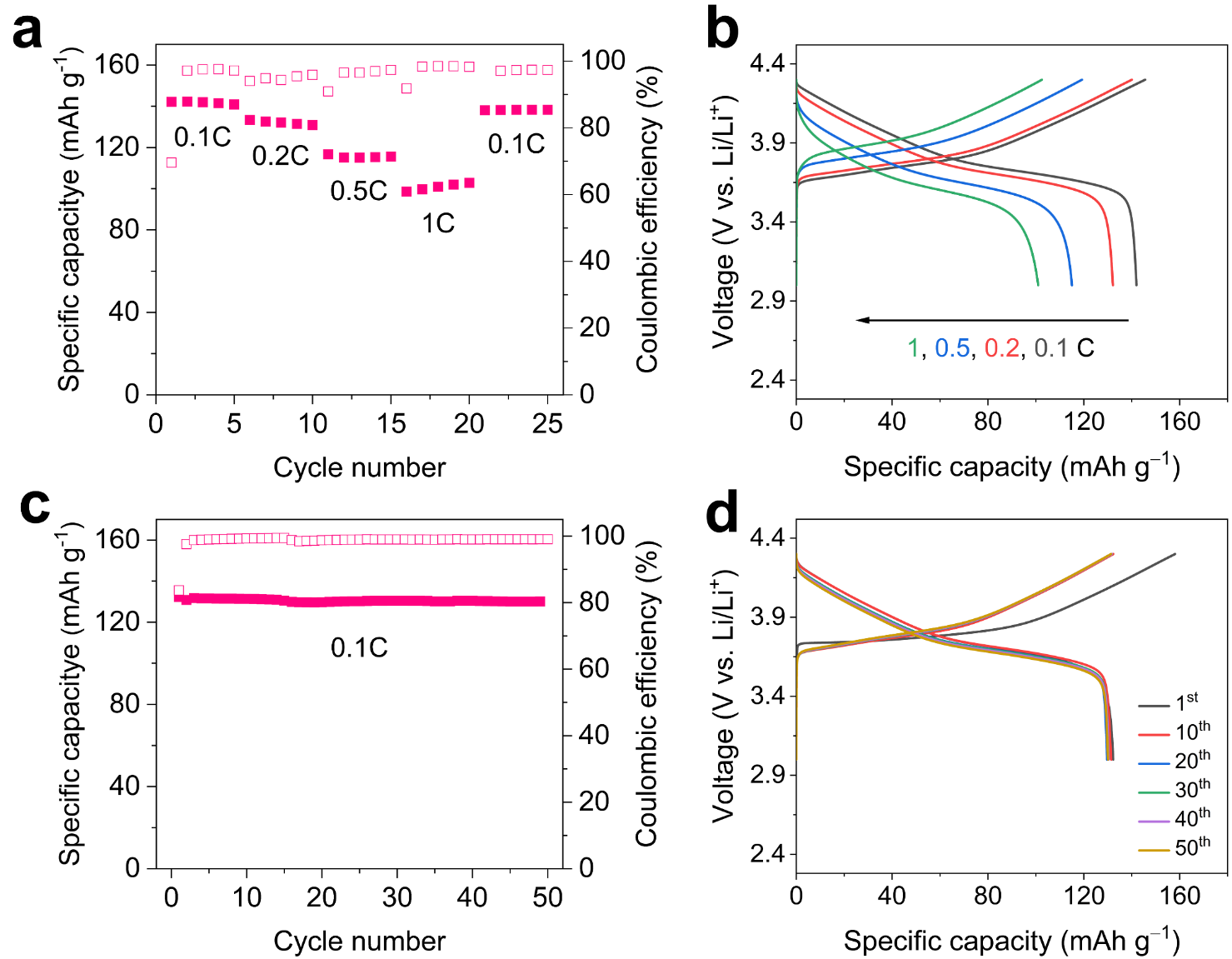


**Fig. S29** (**a**) Rate capability and (**b**) GCD curves of all-solid-state NCM622|PVH-IM|Li full cells at different current densities. (**c**) Cycling stability and (**d**) GCD curves of NCM622|PVH-IM|Li at 0.1 C under 25°C

**Supplementary References**

1. D. Sha, Y. You, R. Hu, X. Cao, Y. Wei et al., Revealing the evolution of doping anions and their impact on K-Ion storage: a case study of Se-doped In_2_S_3_. Energy Storage Mater. **58**, 165–175 (2023). <https://doi.org/10.1016/j.ensm.2023.03.021>
2. X.X. Liu, L. Pan, H. Zhang, P. Yuan, M. Cao et al., Host-guest inversion engineering induced superionic composite solid electrolytes for high-rate solid-state alkali metal batteries. Nanomicro Lett. **17**(1), 190 (2025). <https://doi.org/10.1007/s40820-025-01691-7>
3. T.D. Kühne, M. Iannuzzi, M. Del Ben, V.V. Rybkin, P. Seewald et al., CP2K: an electronic structure and molecular dynamics software package - quickstep: efficient and accurate electronic structure calculations. J. Chem. Phys. **152**(19), 194103 (2020). <https://doi.org/10.1063/5.0007045>
4. J. VandeVondele, M. Krack, F. Mohamed, M. Parrinello, T. Chassaing et al., Quickstep: Fast and accurate density functional calculations using a mixed Gaussian and plane waves approach. Comput. Phys. Commun. **167**(2), 103–128 (2005). <https://doi.org/10.1016/j.cpc.2004.12.014>
5. J. VandeVondele, J. Hutter, Gaussian basis sets for accurate calculations on molecular systems in gas and condensed phases. J. Chem. Phys. **127**(11), 114105 (2007). <https://doi.org/10.1063/1.2770708>
6. S. Goedecker, M. Teter, J. Hutter, Separable dual-space Gaussian pseudopotentials. Phys. Rev. B **54**(3), 1703–1710 (1996). https://doi.org/10.1103/physrevb.54.1703
7. M. Krack, Pseudopotentials for H to Kr optimized for gradient-corrected exchange-correlation functionals. Theor. Chem. Acc. **114**(1), 145–152 (2005). <https://doi.org/10.1007/s00214-005-0655-y>
8. J.P. Perdew, K. Burke, M. Ernzerhof, Generalized gradient approximation made simple. Phys. Rev. Lett. **77**(18), 3865–3868 (1996). <https://doi.org/10.1103/physrevlett.77.3865>
9. S. Grimme, J. Antony, S. Ehrlich, H. Krieg, A consistent and accurate *ab initio* parametrization of density functional dispersion correction (DFT-D) for the 94 elements H-Pu. J. Chem. Phys. **132**(15), 154104 (2010). <https://doi.org/10.1063/1.3382344>
10. S. Grimme, S. Ehrlich, L. Goerigk, Effect of the damping function in dispersion corrected density functional theory. J. Comput. Chem. **32**(7), 1456–1465 (2011). https://doi.org/10.1002/jcc.21759
11. T. Lu, F. Chen, Multiwfn: a multifunctional wavefunction analyzer. J. Comput. Chem. **33**(5), 580–592 (2012). <https://doi.org/10.1002/jcc.22885>
12. A.F. Ferreira, A.J. da Silva, J.A. de Castro, Simulation of the solidification of pure nickel *via* the phase-field method. Mat. Res. **9**(4), 349–356 (2006). <https://doi.org/10.1590/s1516-14392006000400002>
13. L. Chen, H.W. Zhang, L.Y. Liang, Z. Liu, Y. Qi et al., Modulation of dendritic patterns during electrodeposition: a nonlinear phase-field model. J. Power Sources **300**, 376–385 (2015). <https://doi.org/10.1016/j.jpowsour.2015.09.055>
14. M.E. Arguello, M. Gumulya, J. Derksen, R. Utikar, V.M. Calo, Phase-field modeling of planar interface electrodeposition in lithium-metal batteries. J. Energy Storage **50**, 104627 (2022). <https://doi.org/10.1016/j.est.2022.104627>
15. Q. Jian, J. Sun, H. Li, Z. Guo, T. Zhao, Phase-field modeling of zinc dendrites growth in aqueous zinc batteries. Int. J. Heat Mass Transf. **223**, 125252 (2024). <https://doi.org/10.1016/j.ijheatmasstransfer.2024.125252>
